# Genetic variation in the human leukocyte antigen region confers susceptibility to *Clostridioides difficile* infection

**DOI:** 10.1038/s41598-023-45649-4

**Published:** 2023-10-28

**Authors:** Kathleen Ferar, Taryn O. Hall, Dana C. Crawford, Robb Rowley, Benjamin A. Satterfield, Rongling Li, Loren Gragert, Elizabeth W. Karlson, Mariza de Andrade, Iftikhar J. Kullo, Catherine A. McCarty, Abel Kho, M. Geoffrey Hayes, Marylyn D. Ritchie, Paul K. Crane, Daniel B. Mirel, Christopher Carlson, John J. Connolly, Hakon Hakonarson, Andrew T. Crenshaw, David Carrell, Yuan Luo, Ozan Dikilitas, Joshua C. Denny, Gail P. Jarvik, David R. Crosslin

**Affiliations:** 1 Department of Biomedical Informatics and Medical Education, University of Washington, Seattle, WA USA; 2 Optum Genomics, UnitedHealth Group, Minnetonka, MN USA; 3 Department of Population and Quantitative Health Sciences, Cleveland Institute for Computational Biology, Case Western Reserve University, Cleveland, OH USA; 4 Department of Genetics and Genome Sciences, Cleveland Institute for Computational Biology, Case Western Reserve University, Cleveland, OH USA; 5 National Human Genome Research Institute, National Institutes of Health, Bethesda, MD USA; 6 Department of Cardiovascular Diseases, Mayo Clinic, Rochester, MN USA; 7 Division of Biomedical Informatics and Genomics, John W. Deming Department of Medicine, Tulane University School of Medicine, New Orleans, LA USA; 8 Department of Medicine, Brigham and Women’s Hospital, Boston, MA USA; 9 Division of Biomedical Statistics and Informatics, Mayo Clinic, Rochester, MN USA; 10 University of Minnesota Medical School, Duluth, MN USA; 11 Center for Human Genetics, Marshfield Clinic Research Foundation, Marshfield, WI USA; 12 Divisions of General Internal Medicine and Preventive Medicine, Northwestern University, Chicago, IL USA; 13 Division of Endocrinology, Metabolism, and Molecular Medicine, Feinberg School of Medicine, Northwestern University, Chicago, IL USA; 14 Department of Biochemistry and Molecular Biology, Center for Systems Genomics, Pennsylvania State University, University Park, PA USA; 15 Division of General Internal Medicine, University of Washington, Seattle, WA USA; 16 Scilligence Corp., Cambridge, MA USA; 17 Public Health Sciences Division, Fred Hutchinson Cancer Research Center, Seattle, WA USA; 18 Center for Applied Genomics, The Children’s Hospital of Philadelphia, Philadelphia, PA USA; 19 Department of Pediatrics, Children’s Hospital of Philadelphia, Philadelphia, PA USA; 20 The Broad Institute of Harvard-MIT, Cambridge, MA USA; 21 Kaiser Permanente Washington Health Research Institute, Seattle, WA USA; 22 Northwestern University Feinberg School of Medicine, Chicago, IL USA; 23 Department of Biomedical Informatics, Vanderbilt University, Nashville, TN USA; 24 Department of Medicine (Medical Genetics), University of Washington Medical Center, Seattle, WA USA

**Keywords:** MHC class II, Clostridium difficile, Genome-wide association studies, Genetics research

## Abstract

*Clostridioides difficile* (*C. diff.*) infection (CDI) is a leading cause of hospital acquired diarrhea in North America and Europe and a major cause of morbidity and mortality. Known risk factors do not fully explain CDI susceptibility, and genetic susceptibility is suggested by the fact that some patients with colons that are colonized with *C. diff.* do not develop any infection while others develop severe or recurrent infections. To identify common genetic variants associated with CDI, we performed a genome-wide association analysis in 19,861 participants (1349 cases; 18,512 controls) from the Electronic Medical Records and Genomics (eMERGE) Network. Using logistic regression, we found strong evidence for genetic variation in the DRB locus of the MHC (HLA) II region that predisposes individuals to CDI (P > 1.0 × 10^–14^; OR 1.56). Altered transcriptional regulation in the HLA region may play a role in conferring susceptibility to this opportunistic enteric pathogen.

## Introduction

*Clostridioides difficile* (*C. diff.*) infection (CDI), formerly known as *Clostridium difficile* infection, is the leading infectious cause of nosocomial diarrhea in North America and Europe and is associated with a high global burden of disease^1^. Once acquired, this reemerging, Gram-positive, spore-forming bacterium secretes a toxin that causes watery diarrhea, sometimes progressing to severe pseudomembranous colitis, toxic megacolon, and sepsis^2^. In the early 2000s, the emergence of *C. diff.* strain NAP1/BI/027 led to increased incidence, prevalence, morbidity, and mortality associated with CDI^3,4^. This epidemic strain produces more toxin, has a higher resistance to common treatments, and causes more recurrent infections than other common *C. diff.* strains. Despite aggressive antibiotic treatment (e.g. vancomycin, metronidazole, and fidaxomicin) and fecal transplant^5,6^, outcomes of NAP1/BI/027 CDI include significant morbidity across all age groups, 5% mortality in individuals older than 65 years of age, and an estimated $1.1 billion dollars per year in healthcare costs^2^.

Asymptomatic colonization with *C. diff.* is common among patients in healthcare settings, with an estimated prevalence of 3–26% in adults admitted to acute care hospitals and 5–7% in adults at long-term care facilities^7^. Progression from *C. diff.* colonization to acute CDI is generally associated with one or more risk factors^8^, including new exposure to *C. diff.*, older age, hospitalization or nursing home residency, chemotherapy, severe comorbid illness, proton pump inhibitor or immunosuppressant medication use, or prior use of high-risk antibiotics such as fluoroquinolones or cephalosporins^9–11^. Antibiotic use and proton pump inhibitor use are also risk factors for recurrent CDI^12^. Despite having one or more risk factors, some people colonized with *C. diff.* either do not develop CDI or successfully clear an initial infection, while other individuals are burdened by severe and/or recurrent CDI. This differential susceptibility may have a genetic component, given that host genetic variation underlies susceptibility for other infections, including enteric infections such as *Helicobacter pylori*^13^. Identification of host genetic susceptibility loci could yield methods for prevention and/or treatment of this important pathogen^14,15^.

Previous studies have identified candidate risk loci for primary and recurrent CDI in small patient populations using a combination of genetic and clinical data. Apewokin et al.^16^ performed a genome-wide logistic regression analysis of CDI in 646 patients (57 cases; 589 controls) undergoing stem cell transplantation for multiple myeloma, and found several single nucleotide variants (SNVs) in the *RLBP1L1*, *ASPH,* and *P7B* genes that were associated with higher risk of CDI. Shen et al.^17^ identified two alleles in in the extended major histocompatibility complex (MHC; HLA-DRB1*07:01 and HLA-DQA1*02:01) that were associated with a reduction in CDI recurrence among 704 patients who achieved initial clinical cure with bezlotoxumab treatment in the MODIFY clinical trials. Several studies have also suggested that common SNVs in the promoter region of the interleukin-8 (IL-8) gene may confer increased risk for recurrent CDI by altering neutrophil recruitment during disease pathogenesis^18,19^. While these results are collectively suggestive of genetic involvement in CDI risk, the aforementioned studies had small sample sizes and did not always control for major risk factors such as previous antibiotic use or corticosteroid use in their association models. Genome-wide association studies (GWAS) that properly control for known risk factors and include a large number of participants are needed to identify risk loci with sufficient power and reliability. One such study identified 16,464 patients (1160 cases; 15,304 controls) from the Geisinger MyCode cohort^20^ using a *C. diff.* phenotyping algorithm developed by the Electronic Medical Records and Genomics (eMERGE) Network^21^. While no variants reached genome-wide significance in the full case–control dataset, one variant (rs114751021) in the small nucleolar RNA SNORD117 gene, located in the MHC region, reached genome-wide significance in a subset of cases and controls with recent exposure to antibiotics (*P* = 4.50 × 10^–8^; OR 2.42; 587 cases; 3166 controls). Additional validation studies in other large patient cohorts are needed to evaluate the role of genetic factors in CDI risk.

To identify common genetic variants associated with susceptibility to CDI, we performed joint and ancestry-stratified GWAS and human leukocyte antigen (HLA) fine-mapping using phenotypes extracted from electronic medical records (EMRs) of participants aged two years or older from the eMERGE Network. The eMERGE Network is a National Human Genome Research Institute (NHGRI)-funded consortium of twelve study sites across the United States (U.S.) that supports research for furthering the implementation of genomic medicine^22^. At the time of this study, the network included a multi-ethnic cohort of roughly 99,000 U.S. participants with linked genetic and EMR data.

## Results

### Demographics

After all exclusions, there were 1349 cases and 18,512 controls identified via the eMERGE *C. diff.* phenotyping algorithm (Table 1). Approximately 74% of cases and controls self-identified as White, and 19% self-identified as Black or African American. Although older age is a known risk factor for *C. diff.* infection^11^, controls tended to be older than cases (z = 14.37, *P* = 2.20 × 10^–16^), which reflected the patient populations of the participating eMERGE study sites. Controls also tended to have higher BMIs than cases (z = 14.58, *P* = 2.20 × 10^–16^). Cases had slightly higher exposure to Class 1 (high-risk) antibiotics than controls (28% vs. 21%), yet they had much less exposure to Class 2 (moderate risk) antibiotics than controls (11% vs. 26%). More cases received chemotherapy outside of the exclusionary time period than did controls. It is worth noting that while 14 cases were identified from Cincinnati Children’s Medical Hospital, no controls were identified from this site. These cases were 57% female, with a median age of 4.0 (IQR 3.0–12.5) years and a median BMI of 16.09 (IQR 14.90–17.00). Approximately 93% of these cases were of European ancestry (genetically determined) and tended to be at high risk for *C. diff.* infection, with 50% having recent exposure to Class 1 or Class 2 antibiotics and 43% having recent exposure to transplant medications.

**Table 1:** Summary statistics of demographic data and phenotypes for *C. diff* cases and controls selected using the *C. diff* phenotyping algorithm.

N	Case n = 1349	Control n = 18,512	Overall n = 19,861	Case–control differences
Site				
Children’s Hospital of Philadelphia	11% (149)	1.4% (265)	2.1% (414)	**X**^**2**^** = 8.64 (*P* = 3.29 × 10**^**–3**^**)**
Cincinnati Children’s Medical Hospital	1.0% (14)	0.0% (0)	0.1% (14)	**X**^**2**^** = 564.67 (*P* = 2.20 × 10**^**–16**^**)**
Columbia	5.6% (76)	0.5% (88)	0.8% (164)	**X**^**2**^** = 408.56 (*P* = 2.20 × 10**^**–16**^**)**
Geisinger	4.2% (57)	4.9% (899)	4.8% (956)	X^2^ = 1.09 (*P* = 0.30)
Kaiser Permanente/UW	4.2% (57)	11% (2128)	11% (2185)	**X**^**2**^** = 67.87 (*P* = 2.20 × 10**^**–16**^**)**
Mass General Brigham	3.5% (47)	8.8% (1623)	8.4% (1670)	**X**^**2**^** = 45.571 (*P* = 1.47 × 10**^**–11**^**)**
Mayo Clinic	7.2% (97)	17% (3127)	16% (3224)	**X**^**2**^** = 87.03 (*P* = 2.20 × 10**^**–16**^**)**
Marshfield	2.4% (32)	4.7% (861)	4.5% (893)	**X**^**2**^** = 15.207 (*P* = 9.63 × 10**^**–5**^**)**
Mt. Sinai	7.9% (106)	15% (2776)	15% (2882)	**X**^**2**^** = 51.64 (*P* = 3.29 × 10**^**–3**^**)**
Northwestern	5.6% (76)	2.0% (362)	2.2% (438)	**X**^**2**^** = 78.88 (*P* = 2.20 × 10**^**–16**^**)**
Vanderbilt	47% (638)	34% (6383)	35% (7021)	**X**^**2**^** = 90.34 (*P* = 2.20 × 10**^**–16**^**)**
Sex (female)	51% (690)	55% (10,232)	55% (10,922)	**X**^**2**^** = 90.34 (*P* = 2.20 × 10**^**–16**^**)**
Median BMI (kg/m^2^)*	20.8, 25.2, 29.8	24.4, 28.1, 32.9	24.2, 28.0, 32.8	**Z = 14.581 (*P* = 2.20 × 10**^**–16**^**)**
Median age*	39.7, 57.3, 70.0	51.1, 64.9, 76.1	50.4, 64.4, 76.0	**Z = 14.372 (*P* = 2.20 × 10**^**–16**^**)**
Self-identified ancestry				
American Indian or Alaska Native	0.2% (3)	0.2% (40)	0.2% (43)	X^2^ = 0.002 (*P* = 0.96)
Black or African American	15% (196)	19% (3562)	19% (3758)	**X**^**2**^** = 21.75 (*P* = 3.10 × 10**^**–6**^**)**
Asian	0.8% (11)	0.8% (142)	0.8% (153)	X^2^ = 0.04 (*P* = 0.84)
Native Hawaiian or other Pacific Islander	0.07% (1)	0.02% (2)	0.02% (3)	X^2^ = 0.46 (*P* = 0.50)
White	75% (1008)	74% (13,716)	74% (14,724)	X^2^ = 0.26 (*P* = 0.61)
Unknown	9.2% (124)	5.0% (933)	5.3% (1057)	**X**^**2**^** = 43.02 (*P* = 5.42 × 10**^**–11**^**)**
Not reported	0.4% (6)	0.6% (117)	0.6% (123)	X^2^ = 0.72 (*P* = 0.40)
Self-identified ethnicity				
Hispanic or Latino	6.0% (81)	4.8% (895)	4.9% (976)	X^2^ = 3.68 (*P* = 0.06)
Not Hispanic or Latino	88% (1193)	92% (17,120)	92% (18,313)	**X**^**2**^** = 28.62 (*P* = 8.80 × 10**^**–8**^**)**
Unknown	5.6% (75)	2.7% (497)	2.9% (572)	**X**^**2**^** = 37.16 (*P* = 1.09 × 10**^**–9**^**)**
Genetically determined ancestry				
African	17% (235)	21% (3849)	21% (4084)	**X**^**2**^** = 8.75 (*P* = 3.10 × 10**^**–3**^**)**
Asian	2.4% (32)	1.6% (287)	1.6% (319)	**X**^**2**^** = 5.37 (*P* = 2.05 × 10**^**–2**^**)**
European	80% (1082)	78% (14,376)	78% (15,458)	**X**^**2**^** = 4.74 (*P* = 2.95 × 10**^**–2**^**)**
Antibiotic exposure (Within 7–62 days prior to index date)				
High risk	28% (376)	21% (3832)	21% (4208)	**X**^**2**^** = 38.74 (*P* = 4.85 × 10**^**–10**^**)**
Moderate risk	11% (147)	26% (4838)	25% (4985)	**X**^**2**^** = 155.29 (*P* = 2.20 × 10**^**–16**^**)**
Low risk	1.9% (25)	1.5% (284)	1.6% (309)	X^2^ = 0.84 (*P* = 0.36)
No exposure	59% (801)	52% (9558)	52% (10,359)	**X**^**2**^** = 30.233 (*P* = 3.83 × 10**^**–8**^**)**
Cancer (First record to index date + 7 days)	20% (272)	14% (2520)	14% (2792)	**X**^**2**^** = 44.654 (*P* = 2.35 × 10**^**–11**^**)**
Chemotherapy (before 180 days prior to index date, after 7 days following index date)	20% (270)	12% (2263)	13% (2533)	**X**^**2**^** = 68.60 (*P* = 2.20 × 10**^**–16**^**)**
Diabetes mellitus (Ever)	24% (326)	25% (4700)	25% (5026)	X^2^ = 0.99 (*P* = 0.32)
HIV (Ever)	3.0% (44)	2.0% (302)	2.0% (346)	**X**^**2**^** = 19.52 (*P* = 9.94 × 10**^**–6**^**)**
Nursing home status (within 90 days prior to index date)	11% (147)	2.0% (393)	3.0% (540)	**X**^**2**^** = 365.97 (*P* = 2.20 × 10**^**–16**^**)**
Corticosteroid medications (within 21 days prior to index date)	17% (227)	10% (1848)	10% (2075)	**X**^**2**^** = 62.96 (*P* = 2.11 × 10**^**–15**^**)**
Transplant medications (first record to index date + 7 days)	19% (250)	6.0% (1059)	7.0% (1309)	**X**^**2**^** = 335.23 (*P* = 2.20 × 10**^**–16**^**)**

Significant differences between case and control distributions (as determined by chi-squared tests for binary variables and two-sided Z-tests for continuous variables) are shown in bold.

*The three numbers for body mass index (BMI) and age represent the 25th, 50th and 75th quartiles of the distribution.

After finding the intersection of self-reported ancestry and genetically determined ancestry, there were 3700 African participants, 14,620 European participants, and 135 Asian participants. Table 2 summarizes the demographic and phenotype characteristics of the African ancestry cases (n = 192) and controls (n = 3508) and European ancestry cases (n = 988) and controls (n = 13,632), which were used to conduct ancestry-stratified association tests. Cases in the African sample tended to be younger than those in the European sample (median age 50.8 vs. 59.6 years) and had higher rates of diabetes (37% vs. 20%) and HIV (14% vs. 0.8%). There was a higher proportion of female participants among controls in the African sample than in the European sample (66% vs. 52%), and controls in the African sample had higher exposure to high-risk antibiotics (30% vs. 18%) and moderate risk antibiotics (46% vs. 20%) than those in the European sample, as well as higher rates of diabetes (33% vs. 22%). The demographic and risk characteristics of the European sample tended to mirror those of the full study population, but a higher proportion of cases in the European sample identified as not Hispanic or Latino (98% vs. 88%).

**Table 2:** Summary of demographic data and phenotypes for *C. diff* cases and controls in the African ancestry (n = 3700) and European ancestry (n = 14,620) samples.

N	African ancestry (self-ID ∩ GDA) Cases n = 192	African ancestry (self-ID ∩ GDA) Controls n = 3508	European ancestry (self-ID ∩ GDA) Cases n = 988	European ancestry (self-ID ∩ GDA) Controls n = 13,632
Site				
Children’s Hospital of Philadelphia	9.4% (18)	3.5% (124)	8.3% (82)	0.8% (107)
Cincinnati Children’s Medical Hospital	0.5% (1)	0.0% (0)	1.2% (12)	0.0% (0)
Columbia	7.3% (14)	0.4% (14)	2.8% (28)	0.2% (29)
Geisinger	0.0% (1)	0.2% (6)	5.6% (55)	6.5% (891)
Kaiser Permanente/UW	0.0% (1)	2.1% (74)	5.7% (56)	14% (1916)
Mass General Brigham	3.7% (7)	2.6% (92)	3.2% (32)	10% (1384)
Mayo Clinic	0.0% (0)	0.2% (8)	9.3% (92)	22% (3049)
Marshfield	0.0% (0)	0.01% (1)	3.1% (31)	6.3% (853)
Mt. Sinai	27% (52)	50% (1759)	0.7% (7)	1.9% (255)
Northwestern	5.7% (11)	1.9% (67)	6.6% (65)	2.1% (291)
Vanderbilt	45% (87)	39% (1363)	53% (528)	36% (4857)
Sex (female)	57% (109)	66% (2313)	50% (494)	52% (7124)
Median BMI (kg/m^2^)*	21.4, 26.3, 31.4	25.1, 29.7, 35.6	21.0, 25.2, 29.7	24.2, 27.8, 32.4
Median age*	38.5, 50.8, 60.5	46.8, 62.4, 75.5	41.6, 59.6, 72.0	52.1, 65.0, 76.0
Self-identified ethnicity				
Hispanic or Latino	0.00% (0)	0.2% (8)	1.3% (13)	0.6% (86)
Not Hispanic or Latino	100% (192)	99.7% (192)	98% (963)	97% (13,259)
Unknown	0.00% (0)	0.1% (2)	1.2% (12)	2.1% (287)
Antibiotic exposure (within 7–62 days prior to index date)				
High risk	33% (64)	30% (1038)	28% (276)	18% (2461)
Moderate risk	12% (23)	46% (1609)	10% (99)	20% (2665)
Low risk	1.6% (3)	1.3% (46)	2.1% (21)	1.6% (215)
No exposure	53% (102)	23% (815)	60% (592)	61% (8291)
Cancer (First record to index date + 7 days)	15% (29)	11% (391)	23% (223)	15% (2019)
Chemotherapy (Before 180 days prior to index date, after 7 days following index date)	28% (53)	11% (380)	20% (197)	13% (1816)
Diabetes mellitus (ever)	37% (71)	33% (1165)	20% (202)	22% (2978)
HIV (ever)	14% (27)	5.3% (184)	0.8% (8)	0.5% (62)
Nursing home status (within 90 days prior to index date)	11% (21)	2.3% (80)	12% (120)	2.3% (307)
Corticosteroid medications (Within 21 days prior to index date)	18% (34)	13% (455)	18% (174)	9.5% (1293)
Transplant medications (First record to index date + 7 days)	20% (38)	5.4% (190)	17% (169)	6.0% (822)
HLA-DRB haplotypes				
≥ 1 HLA-DRB3, 4 OR 5 gene	98% (188)	97% (3414)	97% (955)	98% (13,336)
≥ 1 HLA-DRB3 gene (DR52)	73% (141)	73% (2548)	57% (559)	61% (8328)
≥ 1 HLA-DRB4 gene (DR53)	31% (60)	33% (1143)	51% (507)	54% (7356)
≥ 1 HLA-DRB5 gene (DR51)	33% (63)	32% (1108)	30% (299)	28% (3831)
No extra DRB gene	2.1% (4)	2.7% (94)	3.3% (33)	2.2% (296)

Ancestry designations reflect the intersection (**∩**) of self-identified ancestry and genetically determined ancestry (GDA).

*The three numbers for body mass index (BMI) and age represent the 25th, 50th and 75th quartiles of the distribution.

### GWAS

**Figure 1:**
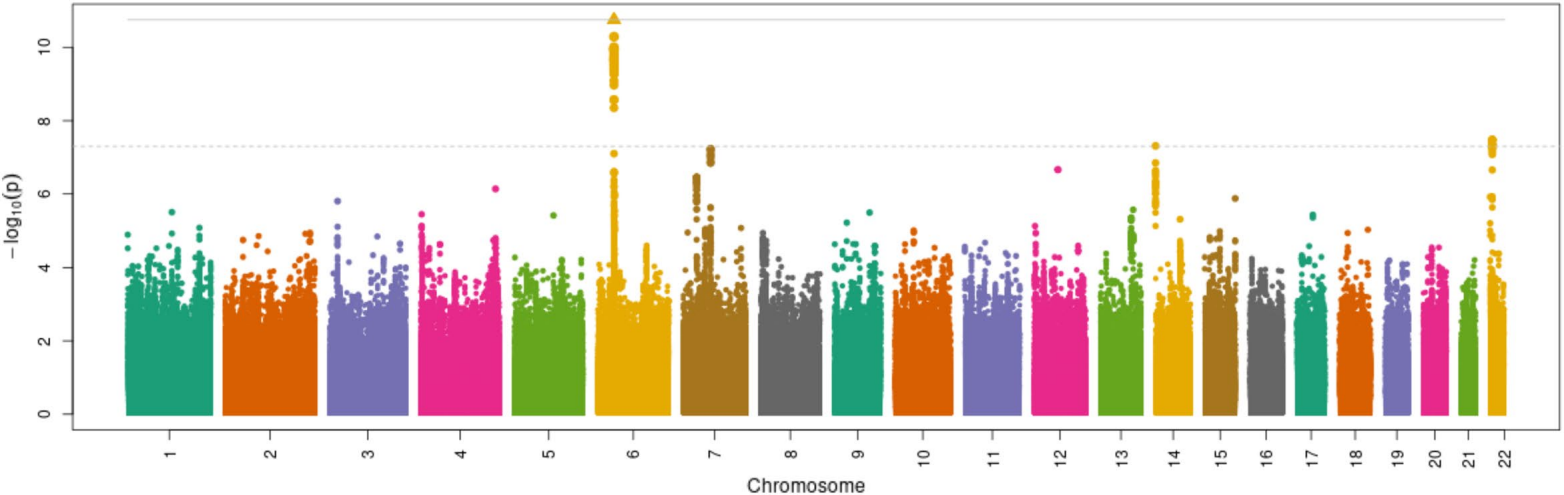
Manhattan plot of *P*-values generated using logistic regression analysis in the European ancestry sample (n = 14,620). An additive model was used to assess the disease susceptibility impact of the minor (coded) allele at each position, while controlling for age, BMI, sex, ancestry, nursing home status, chemotherapy, diabetes, HIV, transplant medications, corticosteroids, and medium or high-risk antibiotic exposure as covariates. Genomic coordinates are displayed along the X-axis, and the negative logarithm of logistic regression *P*-values are displayed on the Y-axis. Each dot represents a SNV in the regression model, with associated *P*-values plotted accordingly, while the triangle represents the most significantly associated SNV. The dotted line represents the negative logarithm of the genome-wide significance threshold (*P* < 5 × 10^–8^). Colors are used to distinguish between SNVs in adjacent chromosomes.

Table 3 summarizes the logistic regression association results that reached genome-wide significance in the combined and European ancestry-only samples, with corresponding summary statistics for those findings in the African ancestry-only sample. A strong association in the human leukocyte antigen (HLA) region was found in the European and joint ancestry samples (Fig. 1, Supplementary Fig. S2) but was not found in the African ancestry sample. The lack of association in the African ancestry sample could be due to either insufficient detection power as a result of small sample size or different haplotype or linkage disequilibrium (LD) structures compared to individuals of European ancestry. Manhattan plots and corresponding QQ plots for the European, joint, and African ancestry GWAS analyses are provided (Supplementary Figs. S1–S5). The five most significantly associated SNVs driving the association in the European sample (rs68148149, *P* = 8.06 × 10^–14^; rs3828840, *P* = 9.96 × 10^–14^; rs35882239, *P* = 8.18 × 10^–12^; rs71534541, P = 5.12 × 10^–11^; rs35222480, P = 9.88 × 10^–11^) mapped to the intergenic region between the *HLA-DRB5* and *HLA-DRB1* genes in the beta block of the MHC Class II region. Three of the five most significant SNVs (rs3828840, rs35882239, and rs35222480), with minor allele frequencies (MAFs) of 0.17, 0.17, and 0.20, respectively, also mapped to the 3ʹ end of the HLA-DRB6 pseudogene. A review of the NHGRI-European Bioinformatics Institute (NHGRI-EBI) GWAS Catalog^23^ and dbSNP^24^ revealed that rs3828840 has been previously associated with multiple sclerosis, an autoimmune inflammatory disease that impacts the central nervous system^25^.

**Table 3:** Index SNV results from logistic regression-based genome wide analysis for joint ancestry (n = 19,861), European ancestry (n = 14,620), and African ancestry (n = 3700) samples.

Chr	SNV	Ref	Alt	CA	BP	Joint CAF (n = 19,861)	Logistic joint P-value	EUR CAF (n = 14,620)	Logistic EUR P-value	Logistic EUR SNV-controlled P-value	AFR CAF (n = 3700)	Logistic AFR P-value
							OR (95% CI)		OR (95% CI)	OR (95% CI)		OR (95% CI)
							Beta		Beta	Beta		Beta
6	rs68148149	C	A	C	32,511,725	0.17	**6.83 × 10**^**–9**^	0.17	**8.06 × 10**^**–14**^	0	0.18	7.2 × 10^–1^
							**1.36 (1.06–1.74)**		**1.56 (1.13–2.15)**	0		0.95 (0.80–1.13)
							**0.13**		**0.2**	0		-0.02
6	rs3828840	T	C	T	32,520,907	0.17	**8.42 × 10**^**–9**^	0.17	**9.96 × 10**^**–14**^	0	0.18	7.1 × 10^–1^
							**1.36 (1.06–1.74)**		**1.56 (1.13–2.15)**	0		0.95 (0.79–1.13)
							**0.13**		**0.2**	0		-0.02
6	rs35882239	A	G	A	32,522,576	0.2	**1.32 × 10**^**–8**^	0.21	**8.18 × 10**^**–12**^	9.80 × 10–1	0.2	6.7 × 10^–1^
							**1.34 (1.05–1.70)**		**1.49 (1.10–2.00)**	1.00 (1.00–1.00)		0.94 (0.78–1.13)
							**0.13**		**0.17**	0		-0.03
6	rs71534541	C	T	C	32,513,076	0.08	7.98 × 10^–7^	0.07	**5.12 × 10–11**	2.30 × 10^–1^	0.1	8.2 × 10^–1^
							1.38 (1.04–1.80)		**1.62 (1.12–2.33)**	1.15 (0.90–1.46)		0.96 (0.81–1.14)
							0.14		**0.21**	0.06		-0.02
6	rs35222480	A	T	A	32,522,813	0.08	8.41 × 10^–7^	0.08	**9.88 × 10–11**	2.20 × 10^–1^	0.1	5.0 × 10^–1^
							1.37 (1.04–1.80)		**1.59 (1.11–2.26)**	1.14 (0.90–1.44)		0.89 (0.66–1.19)
							0.14		**0.2**	0.06		-0.05
6	rs116603449	C	T	T	32,595,194	0.21	**6.59 × 10**^**–9**^	0.21	**5.42 × 10–10**	**4.54 × 10**^**–9**^	0.22	8.73 × 10^–2^
							**1.31 (1.05–1.62)**		**1.39 (1.07–1.80)**	**1.37 (1.06–1.77)**		1.24 (0.90–1.70)
							**0.12**		**0.14**	**0.14**		0.09
6	rs9270896	A	G	G	32,571,876	0.41	1.27 × 10^–5^	0.42	1.21 × 10^–5^	**6.09 × 10**^**–9**^	0.33	3.96 × 10^–2^
							1.19 (1.01–1.40)		1.22 (1.01–1.47)	**1.32 (1.05–1.65)**		1.26 (0.92–1.74)
							0.08		0.09	**0.12**		0.1
6	rs9270894	A	G	G	32,571,872	0.26	1.17 × 10^–5^	0.24	1.66 × 10^–6^	**1.12 × 10–8**	0.32	1.16 × 10^–1^
							1.22 (1.01–1.47)		1.29 (1.03–1.63)	**1.37 (1.06–1.77)**		1.20 (1.90–1.58)
							0.09		0.11	**0.14**		0.08
6	rs9270895	C	T	T	32,571,873	0.45	5.95 × 10^–5^	0.44	5.39 × 10^–5^	**2.32 × 10–8**	0.42	3.54 × 10^–2^
							1.17 (1.00–1.37)		1.21 (1.00–1.45)	**1.31 (1.05–1.64)**		1.26 (0.92–1.73)
							0.07		0.08	**0.12**		0.1
6	rs618095	G	A	A	32,574,736	0.28	5.05 × 10^–7^	0.25	2.69 × 10^–6^	**3.71 × 10–8**	0.36	1.19 × 10^–2^
							1.26 (1.03–1.53)		1.29 (1.02–1.62)	**1.35 (1.05–1.73)**		1.32 (0.94–1.87)
							0.1		0.11	**0.13**		0.12

An additive model was used to assess the disease susceptibility impact of the minor (coded) allele at each position, while controlling for age, BMI, sex, ancestry, nursing home status, chemotherapy, diabetes, HIV, transplant medications, corticosteroids, and medium or high-risk antibiotic exposure as covariates.

*Chr* chromosome, *SNV* single nucleotide variant, *Ref* reference allele, *Alt* alternate allele, *CA* coded allele, *BP* base pair, *CAF* coded allele frequency, *OR* odds ratio.

Results meeting the genome-wide significance threshold (*P* < 5 × 10^–8^) are displayed in bold.

**Figure 2:**
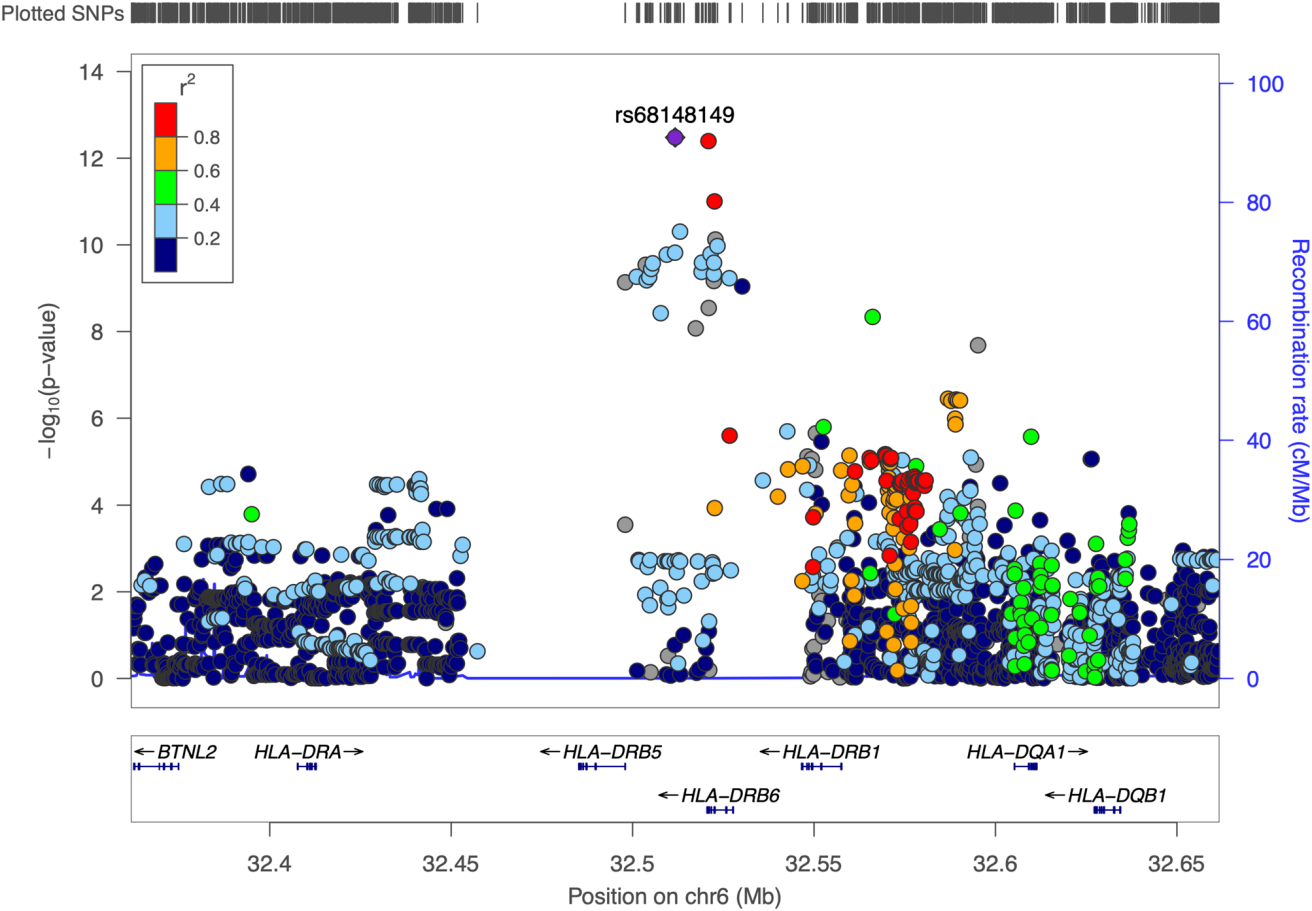
Regional LD plot of SNVs evaluated in the European-ancestry logistic regression analysis, using the European 1000 Genomes superpopulation as a reference group. Genomic coordinates spanning the HLA-DRB region and surrounding genes are shown on the X-axis in both subplots. Negative logarithms of *P*-values from the European-ancestry logistic regression analysis are shown on the Y-axis in the upper sublot, and annotated gene transcripts are distributed along the Y-axis in the lower subplot. Each dot represents a SNV in the regression model, with associated *P*-values plotted accordingly. SNVs in highest LD with reference to the index SNV (rs68148149) are colored in red. The LD plot was generated with the LocusZoom^68^ tool using default parameters and the 1000 Genomes Project 2014 EUR reference panel.

Given the well-known presence of high LD within the HLA region^26^, a regional LD plot with reference to the index SNV (rs68148149) was generated using *P*-values from the European logistic regression analysis and using the 2014 1000 Genomes European superpopulation as a reference group (Fig. 2). This step was taken to assess the possibility that variants other than the index SNV might better explain disease association in terms of functional impact. While the second two most significant SNVs were in high LD with the index SNV (R^2^ > 0.8), the index SNV had the highest regulatory potential among the most significantly associated SNVs, as annotated by RegulomeDB^27^. To assess the possibility that the lack of disease association in the African ancestry sample is a result of different regional LD structures, a regional LD plot with reference to the index SNV was generated using the 1000 Genomes African superpopulation as a reference (Supplementary Fig. S6). The second two most significant SNVs in the European-ancestry sample were also in high LD with the index SNV in the African-ancestry superpopulation, but higher LD was observed with more SNVs in the HLA-DRB1/5 intergenic region in the African superpopulation (R^2^ > 0.4) than in the European superpopulation (R^2^ > 0.2). On the other hand, lower LD was observed with SNVs in the region spanning HLA-DRB1 and HLA-DQA1 in the African superpopulation (R^2^ > 0.6) than in the European superpopulation (R^2^ > 0.8). Differences in regional LD patterns between the European-ancestry and African-ancestry samples could therefore have contributed to the observed differences in gene-disease association patterns, in addition to insufficient detection power.

A follow-up GWAS using the index SNV as a covariate revealed several new SNVs associated at genome-wide significance (rs116603449, *P* = 4.54 × 10^–9^; rs9270896, *P* = 6.09 × 10^–9^; rs9270894, *P* = 1.12 × 10^–8^; rs9270895, *P* = 2.32 × 10^–8^; rs618095, *P* = 3.71 × 10^–8^) (Table 3, Supplementary Figs. S7, S8). While suggestive peaks were observed in chromosomes 14 and 22 using the unadjusted model, the elimination of these peaks in models that included the genome-wide significant index SNVs suggests that they were spuriously associated with the tagged region in chromosome 6. However, no SNVs of interest on chromosomes 14 or 22 were in high LD with any of the index SNVs on chromosome 6, therefore the nature of the association remains unknown.

### HLA association analyses

All 14,620 European ancestry participants had high quality imputed HLA genotypes available for association analyses. Table 2 summarizes the number of participants in each ancestry stratified case–control group possessing at least one *HLA-DRB3*, *4* and/or *5* gene (corresponding to haplotype families HLA-DR52, 53 and 51, respectively)^28^ (Supplementary Fig. S11). The most significant SNVs from the GWAS reached genome-wide significance among individuals with at least one *DRB3*, *4* or *5* genes collectively (rs68148149, *P* = 1.26 × 10^–13^; rs3828840, *P* = 1.49 × 10^–13^; rs35882239, *P* = 2.37 × 10^–11^; rs71534541, *P* = 1.67 × 10^–11^; rs35222480, *P* = 3.17 × 10^–11^), and among individuals with at least one *DRB5* gene only, or DR51 haplotype carriers (rs68148149, *P* = 1.55 × 10^–11^; rs3828840, *P* = 1.72 × 10^–11^; rs35882239, *P* = 2.62 × 10^–10^; rs71534541, *P* = 1.56 × 10^–11^; rs35222480, *P* = 4.68 × 10^–11^) (Table 4, Supplementary Fig. S9). Among DR51 haplotype carriers, the most significantly associated SNVs only reach genome-wide significance among carriers of the DR15 haplotype (rs68148149, *P* = 2.08 × 10^–11^; rs3828840, *P* = 2.27 × 10^–11^; rs35882239, *P* = 4.14 × 10^–10^; rs71534541, *P* = 1.75 × 10^–12^; rs35222480, *P* = 5.81 × 10^–12^), and more specifically, carriers of the HLA-DRB1*15:01 allele (rs68148149, *P* = 7.45 × 10^–11^; rs3828840, *P* = 8.11 × 10^–11^; rs35882239, *P* = 1.42 × 10^–9^; rs71534541, *P* = 7.37 × 10^–12^; rs35222480, *P* = 1.43 × 10^–11^). No SNVs reached genome-wide significance among participants with at least one *DRB3* or *DRB4* gene only, suggesting that the HLA-DR51 haplotype in combination with variants in the HLA-DRB1/5 intergenic region may singularly drive genetic risk for CDI in the European ancestry population. However, examining the risk allele frequencies of the index SNV (rs68148149) in cases and controls across DR51, DR52, and DR53 haplotype-enriched groups showed that the risk allele frequency was higher in European-ancestry cases than controls in all haplotype groups, suggesting that the SNV may indeed drive risk in all HLA-DR haplotype groups but that the low frequency in the DR52 and DR53 haplotype groups limits the power to detect the association in these groups (Supplementary Fig. S12). The same pattern was not observed in African-ancestry cases and controls, indicating that haplotype differences between ancestry groups may indeed play a role in differentially conferring risk.

**Table 4:** Index SNV results from logistic regression-based analysis of the HLA region in European samples enriched for each HLA-DRB haplotype or haplotype family: DR51, DR52, DR53, DR15, DRB1*15:01, and any of the above.

Chr	SNV	Ref	Alt	CA	BP	DR51(+), DR52(+), or DR53(+)		DR51(+)		DR52(+)		DR53(+)		DR15(+)		DRB1*15:01(+)	
						CAF (n = 14,291)	Logistic P-value	CAF (n = 4130)	Logistic P-value	CAF (n = 8887)	Logistic P-value	CAF (n = 7863)	Logistic P-value	CAF (n = 3791)	Logistic P-value	CAF (n = 3608)	Logistic P-value
							OR (95% CI)		OR (95% CI)		OR (95% CI)		OR (95% CI)		OR (95% CI)		OR (95% CI)
							Beta		Beta		Beta		Beta		Beta		Beta
6	rs68148149	C	A	C	32,511,725	0.12	**1.26 × 10**^**–13**^	0.32	**1.55 × 10**^**–11**^	0.08	1.50 × 10^–4^	0.08	5.02 × 10^–7^	0.32	**2.08 × 10**^**–11**^	0.32	**7.54 × 10**^**–11**^
							**1.59 (1.14–2.22)**		**1.97 (1.18–3.29)**		1.53 (1.00–2.34)		1.78 (1.07–2.93)		**2.01 (1.18–3.41)**		**2.00 (1.17–3.41)**
							**0.2**		**0.29**		0.18		0.25		**0.3**		**0.3**
6	rs3828840	T	C	T	32,520,907	0.12	**1.49 × 10**^**–12**^	0.32	**1.72 × 10**^**–11**^	0.08	1.70 × 10^–4^	0.08	5.25 × 10^–7^	0.32	**2.27 × 10**^**–11**^	0.32	**8.11 × 10**^**–11**^
							**1.59 (1.14–2.22)**		**1.97 (1.18–3.29)**		1.52 (1.00–2.33)		1.78 (1.07–2.93)		**2.01 (1.18–3.41)**		**2.00 (1.17–3.40)**
							**0.2**		**0.29**		0.18		0.25		**0.3**		**0.3**
6	rs35882239	A	G	A	32,522,576	0.15	**2.37 × 10**^**–11**^	0.37	**2.62 × 10**^**–10**^	0.1	1.20 × 10^–3^	0.1	4.02 × 10^–5^	0.38	**4.14 × 10**^**–10**^	0.37	**1.42 × 10**^**–9**^
							**1.50 (1.10–2.04)**		**1.95 (1.16–3.29)**		1.42 (0.97–2.07)		1.56 (1.01–2.40)		**1.99 (1.16–3.40)**		**1.97 (1.15–3.38)**
							**0.18**		**0.29**		0.15		0.19		**0.3**		**0.3**
6	rs71534541	C	T	C	32,513,076	0.08	**1.67 × 10**^**–11**^	0.26	**1.56 × 10**^**–11**^	0.04	7.80 × 10^–3^	0.05	9.94 × 10^–6^	0.28	**1.75 × 10**^**–12**^	0.28	**7.37 × 10**^**–12**^
							**1.64 (1.13–2.38)**		**2.069 (1.19–3.58)**		1.45 (0.93–2.27)		1.82 (1.04–3.16)		**2.25 (1.22–3.91)**		**2.23 (1.22–4.05)**
							**0.21**		**0.32**		0.16		0.26		**0.35**		**0.35**
6	rs35222480	A	T	A	32,522,813	0.08	**3.17 × 10**^**–11**^	0.27	**4.68 × 10**^**–11**^	0.05	1.20 × 10^–2^	0.06	9.33 × 10^–6^	0.29	**5.81 × 10**^**–12**^	0.29	**1.43 × 10**^**–11**^
							**1.61 (1.12–2.31)**		**2.01 (1.18–3.44)**		1.41 (0.92–2.16)		1.73 (1.04–2.87)		**2.18 (1.22–3.91)**		**2.18 (1.21–3.93)**
							**0.21**		**0.3**		0.15		0.24		**0.34**		**0.34**

An additive model was used to assess the disease susceptibility impact of the minor (coded) allele at each position in the genomic region that yielded highly associated SNVs in the genome-wide analysis (chr6:32,400,001–32,600,000). Age, BMI, sex, ancestry, nursing home status, chemotherapy, diabetes, HIV, transplant medications, corticosteroids, and medium or high-risk antibiotic exposure were included as covariates in the model.

*Chr* chromosome, *SNV* single nucleotide variant, *Ref* reference allele, *Alt* alternate allele, *CA* coded allele, *BP* base pair, *CAF* coded allele frequency, *OR* odds ratio.

Results meeting the genome-wide significance threshold (*P* < 5 × 10^–8^) are displayed in bold.

To assess the possibility that one or more HLA alleles themselves were driving the risk association in the European ancestry sample, rather than the most significantly associated SNVs identified in the GWAS, we performed a separate logistic regression analysis using the HIBAG-imputed HLA genotypes in the European ancestry sample. None of the imputed HLA alleles reached genome-wide significance. Using the classical HLA tags identified by de Bakker et al.^29^ and the NCI LDMatrix tool^30^, it was also confirmed that none of the GWAS-identified SNVs were in high LD (R^2^ > 0.5) with any classical HLA alleles in either the European ancestry or African ancestry 1000 Genomes superpopulations. The index SNV was in moderate LD with the tag SNV for the DRB1*15:01-DRB5*01:01 haplotype in the European ancestry superpopulation (rs3135388; R^2^ = 0.186) and low LD with the tag SNV in the African ancestry superpopulation (rs443623; R^2^ = 0.002).

## Discussion

Using a robust EMR-based phenotyping algorithm, we identified a large, multi-institutional corpus of patients with a history of at least one episode of CDI and controls without CDI. Our results suggest that genetic variation in the *(HLA-)DRB* locus of the HLA region may increase risk of infection in European ancestry populations. In this study, European participants who possessed the minor allele among the most significantly associated SNVs had 56% greater odds of having at least one episode of CDI. As the key beta-subunits of MHC Class II surface receptors on antigen presenting cells (APCs), the proteins encoded by *DRB* genes play a critical role in stimulating the host adaptive immune response against foreign peptides and are therefore excellent candidates for future studies of host immunity to *C. diff.*^31^.

The MHC (HLA) Class I and II loci are among the most polymorphic coding regions in the human genome, and *DRB* genes are particularly variable in copy number and combination. Although there is only one monomorphic *DRA* gene per (HLA-)DR haplotype, there are five common DR haplotype families composed of different combinations of protein coding *DRB* genes (*DRB1, DRB3, DRB4* and *DRB5*) and pseudogenes (*DRB2, DRB6, DRB8* and *DRB9*)^28^. *DRB1* is present in all haplotypes, but any given individual may have as few as two protein coding *DRB* genes (2 copies of *DRB1*), or as many as four genes (2 copies of *DRB1* + 1 or 2 copies of *DRB3*, *4* or *5*) between homologs. The unique combination of *DRB* genes on each haplotype is remarkably conserved and has been maintained in ancestral DNA since before the divergence of human and gorilla lineages over five million years ago^32^. Although having a diverse set of MHC II molecules may confer a selective advantage against infection^33^, each additional *DRB* gene is nonetheless susceptible to intragenic and/or regulatory mutations in the highly polymorphic HLA region and may paradoxically increase susceptibility to other diseases. In the case of gastrointestinal infections, protective effects of the DRB1*04:05 allele against enteric infection caused by *Salmonella typhi* or *Salmonella paratyphi* have been observed in Vietnamese and Nepalese patients^34^. Conversely, the *DRB1* gene has also been implicated in increasing host susceptibility to a number of inflammatory diseases, including Crohn’s disease, type I diabetes mellitus, rheumatoid arthritis, multiple sclerosis (MS), ulcerative colitis and Alzheimer’s disease, primarily in European populations^35–40^.

Haplotype effects appear to play a critical role in conferring risk for CDI. In this study, the risk association only reached genome-wide significance in individuals carrying at least one copy of the DRB1*15:01-DRB5*01:01 haplotype^41^, and individuals in this group had 200% higher odds of developing CDI on average. These results indicate that the DRB1*15:01-DRB5*01:01 haplotype is involved in conferring CDI risk among individuals with common genetic variants in the tagged *DRB1-DRB5* intergenic region (Supplementary Fig. S10). This haplotype is most strongly associated with susceptibility to multiple sclerosis^42–45^, but has also been associated with susceptibility to other autoimmune conditions including anti-glomerular basement membrane disease in European ancestry populations^46,47^, and both systemic lupus erythematosus and adult onset Still’s disease in Japanese populations^48^.

One possible explanation for increased CDI risk among these individuals is that differential MHC II gene expression impacts the baseline composition of their gut microbiota, thereby influencing colonization resistance to opportunistic enteric pathogens like *C. diff.* Secretory Immunoglobulin A (IgA) antibodies play an essential role in shaping an individual’s gut microbial community and maintaining a homeostatic balance of microbes within the mucosal immune system^49^, and the interactions between APCs and CD4+ T-follicular helper (Tfh) cells are key to driving the production of IgA by plasma cells^50^. Studies in mouse models have previously demonstrated that MHC II polymorphisms directly affect antibody-mediated microbiota composition, and that the unique microbial communities formed under the influence of different MHC genotypes can impact an organism’s susceptibility to opportunistic pathogens like *Salmonella enterica typhimurium* when treated with antibiotics^51,52^. Understanding the unique interactions between commensal microbe antigens presented by APCs, the MHC II molecules encoded by the DRB1*15:01-DRB5*01:01 haplotype, and Tfh cells may provide valuable insights into how host genetics impact the composition of gut microbial communities in individuals susceptible to enteric infection, compared with those who are resistant to infection.

Alternatively, increased CDI risk among these individuals may be driven by differential T-cell mediated responses to the TcdA and TcdB toxins produced by *C. diff.* bacteria. In addition to sculpting the host microbiota, high affinity IgA helps to neutralize bacterial toxins^53^. Unique interactions between T-cells and *C. diff.* toxins specifically bound by DRB1*15:01-DRB5*01:01 MHC II molecules may impact the host anti-toxin IgA response differently than other T-cell-MHC II interactions, thus influencing the host’s ability to clear circulating toxins. Recent Phase III, placebo-controlled clinical trials of the monoclonal antibody treatments actoxumab (anti-TcdA) and bezlotoxumab (anti-TcdB) showed that TcdB toxin neutralization alone could decrease CDI recurrence by 38% among patients receiving standard antibiotic therapy for initial or recurrent CDI^54^. Naturally occurring anti-TcdB antibodies in the placebo group also conferred protection against recurrent CDI, recapitulating the importance of neutralizing TcdB in controlling infection^55^. However, other studies have failed to replicate these results when comparing healthy controls with CDI patients, suggesting that anti-toxin antibody concentrations may not fully explain susceptibility to initial and/or recurrent infection^56^.

Although the MHC II region is strongly associated with CDI in this study, the SNVs that confer risk are neither located in coding regions, nor in high LD with SNVs in coding regions, suggesting that the mechanism for altered gene expression may be regulatory. One possible mechanism for altered expression of the DRB1*15:01-DRB5*01:01 haplotype is allele-specific DNA methylation of the *DRB1* and/or *DRB5* regulatory regions, given that that targeted bisulfite sequencing has previously identified the *DRB1*-*DRB5* intergenic space as a differentially methylated region^57^. Disruptions to normal DNA methylation patterns, and to resulting gene expression, have been known to modulate susceptibility to a number of human diseases^58^. For example, in the case of DRB1*15:01-DRB5*01:01-associated multiple sclerosis, DNA hypermethylation in exon 2 of *DRB1* confers protection against the major risk allele and is driven by several SNVs in high LD with one another that overlap with CpG sites^59^. It is possible that disrupted methylation patterns at or near the regulatory regions of DRB1*15:01 and/or DRB5*01:01 also contribute to differential expression of these MHC II proteins, thus impacting the landscape of the host adaptive immune response via microbiome-mediated and/or toxin-mediated mechanisms. Additional gene expression analyses, such as expression quantitative trait loci (eQTL) analysis, could be used to explore whether the top SNVs regulate expression levels of nearby genes.

This study has several important limitations. First, sample size and statistical power were severely limited among non-European ancestry samples, which may have contributed to the lack of significant associations in the African ancestry analyses. It is also possible that within the European sample, the comparatively low frequency of the risk allele in the DR52 and DR53 haplotype groups, compared to DR51, limited the power to detect a true risk association in other DR haplotype groups. Second, replicate studies are needed to confirm the identified association. However, the large, multi-site biobank of linked EMR and genotype data used in this study supports the replicability and reliability of these results, and future association studies would benefit immensely from these types of biobanks. While the gene associations in this study do not align exactly with those identified in the previous *C. diff.* GWAS conducted by Li et al. using the MyCode cohort, they do support the hypothesis that immune molecules encoded within the MHC region are involved in CDI pathogenesis. Third, *C. diff.* cases were not stratified by primary and recurrent CDI, and it is possible that the genetic variants driving pathogenesis are different between these two forms of infection. For example, Shen et al. identified alleles in *DRB1* and *DQA1* that were different from those identified in this study and were protective against CDI recurrence, suggesting that the genetic factors involved in initial vs. recurrent infection could be distinct from one another. Fourth, the length and severity of infection were not considered in the current study, but future analyses would benefit from continuous trait regression analyses to identify genetic variants associated with increased CDI length and/or severity, rather than susceptibility. Additionally, *C. diff.* cases in this study included individuals with a positive antigen test as their only criterion for infection. The *C. diff.* antigen test cannot accurately distinguish between toxigenic and non-toxigenic strains and may falsely identify asymptomatic carriers as *C. diff.* cases. Finally, the specific toxigenic ribotype that each case was exposed to was not included in the analysis, and it is possible that different *C. diff.* ribotypes are associated with different genetically determined host responses.

Our findings suggest that genetic variation in the MHC II locus of the HLA region drives susceptibility to CDI and highlights the importance of the adaptive immune response in combating opportunistic pathogens. To better understand how host genetics might confer microbiome-mediated risk for opportunistic enteric infections, future studies should explore the mechanisms of interaction between commensal microbe antigens presented by APCs and the MHC II molecules encoded by the DRB1*15:01-DRB5*01:01 haplotype. Interactions between DRB1*15:01-DRB5*01:01 MHC II molecules, *C. diff.* exotoxins and T-cells may alternately play a critical role in CDI pathogenesis, and additional work is needed to understand whether and how the host IgA response is differentially impacted by the combined effects of haplotype and transcriptional modifications. Finally, future work should address the possibility that allele-specific DNA methylation is a driver of epigenetic transcriptional regulation of the *DRB1* and/or *DRB5* genes. If this mechanism is experimentally validated, therapeutics that modulate MHC II molecule transcription levels could potentially be developed to decrease the incidence of CDI among individuals who carry the risk genotype.

## Methods

### Participants

Cases and controls were selected from among the ~ 99,000 participants of the eMERGE Network. Participating sites included the following: 1. The Children’s Hospital of Philadelphia, Philadelphia, PA; 2. Cincinnati Children’s Medical Hospital, Cincinnati, OH; 3. Columbia University, New York, NY; 4. Geisinger, Danville, PA; 5. Mass General Brigham, Boston, MA; 6. Kaiser Permanente Washington (formerly Group Health Cooperative) and University of Washington partnership, Seattle, WA; 7. Marshfield Clinic, Marshfield, WI; 8. Mayo Clinic, Rochester, MN; 9. Meharry Medical College, Nashville, TN; 10. Mount Sinai, New York, NY; 11. Northwestern University, Evanston, IL; and 12. Vanderbilt University, Nashville, TN. Informed consent was obtained from participants by each eMERGE site. The eMERGE study was approved by each participating site’s institutional review board, and all methods were performed in accordance with the relevant guidelines and regulations at each institution.

### Case–control selection using *Clostridioides difficile* phenotyping algorithm

**Figure 3:**
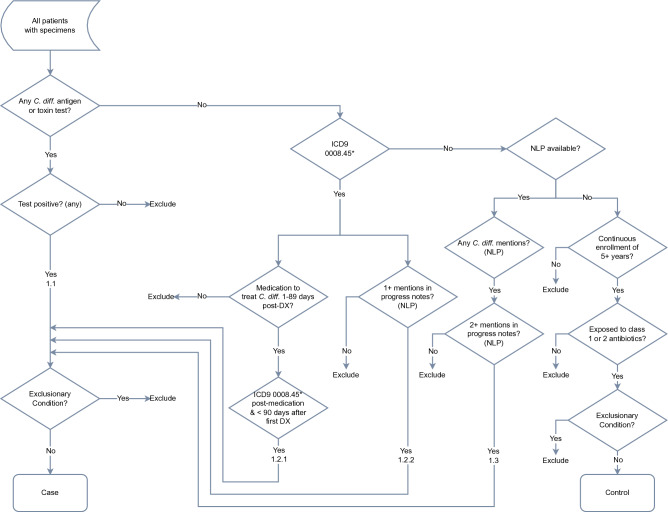
eMERGE *Clostridiodes difficile* phenotyping algorithm flowchart.

*Clostridioides difficile* cases and controls were selected using a variety of information contained in the EMR, including International Classification of Disease (ICD) Clinical Modification (CM) codes 9th and 10th editions, lab and medication data, and clinician progress notes. The *C. diff.* phenotyping algorithm used in this study was designed collaboratively by the University of Washington, Group Health and Vanderbilt as part of the eMERGE Network and was published in the Phenotyping KnowledgeBase (PheKB) in 2012^60,61^. Case/control selection and exclusion criteria are depicted as a flowchart in Fig. 3.

For participants aged two years or older, there were four combinations of EMR data considered for case selection. First, individuals with a positive *C. diff.* antigen or toxin test were selected. Second, those with one or more inpatient or outpatient diagnoses of *C. diff.* (ICD-9-CM code 008.45; ICD-10-CM code A047), followed by one or more days of medication for treatment (metronidazole, oral vancomycin, fidaxomicin, or linezolid), followed by another inpatient or outpatient *C. diff.* diagnosis code, were selected. Third, individuals with at least one *C. diff.* ICD-CM code combined with at least one affirmative mention (unqualified by negation, uncertainty, or historical reference) of *C. diff.* infection in a clinical progress note as identified through natural language processing (NLP), were selected. The *C. diff.* mentions used by the NLP algorithm are listed in Supplementary Table S1. Finally, individuals with two or more affirmative mentions of *C. diff.* infection on separate calendar days in clinical progress notes, identified by NLP, were selected. To exclude severely immune-compromised participants from the test population, participants meeting one of the four above criteria were excluded from being cases if they had a diagnosis of bone marrow cancer in the 2-year period prior to their *C. diff.* case index date (i.e., the first positive lab test, diagnosis code or progress note mention), or within 7 days following their index date. Participants were also excluded from being cases if they had received chemotherapy in the 180-day period prior to their *C. diff.* index date, or within 7 days following their index date. Using these criteria, 1598 cases were selected.

Controls were selected from eMERGE participants two years of age or older who had no known test for and no diagnosis codes for *C. diff.* in their records. Since *C. diff.* toxin tests have sensitivities ranging from 60 to 70%^62^, a single test does not rule out disease, and multiple tests could signal a concern that disease exists. Additionally, controls must have had at least one hospital admission with a prior exposure to a high-or moderate-risk antibiotic (Supplementary Table S2) in the 7 to 62-day period before admission. Alternatively, they must have had exposure to a high or moderate-risk antibiotic and had 5 or more years of documented clinical visits following exposure with no mention of *C. diff.* infection in their progress notes. Participants meeting the control criteria were excluded if they had chemotherapy or bone marrow cancer in the 180-day period prior to the *C diff.* control index date (i.e., the earliest hospital admission with antibiotic exposure or earliest antibiotic exposure with 5 years of follow-up), or within seven days following the index date. These criteria resulted in the selection of 23,061 eMERGE participants as controls.

We excluded 202 cases and 2723 controls that were missing genotype data. An additional 31 cases and 889 controls were excluded because the genotype imputation quality failed to meet our quality control (QC) threshold (mean R^2^ > 0.3)^63^.

Cryptic relatedness was assessed in all participants by calculating the probabilities of sharing alleles identical by descent (IBD), where Z0 is the probability of sharing zero alleles IBD and Z1 is the probability of sharing one allele IBD. Families were constructed when sample pairs had Z0 < 0.83 and Z1 > 0.1^63^. When study participants were found to be in the same family, we prioritized the inclusion of cases. In situations where two or more cases or two or more controls were found to be in the same family, one participant was selected at random, and the others were excluded. For participants selected via the *C. diff.* phenotyping algorithm, 9 cases and 937 controls were excluded due to cryptic relatedness. Two-sample Z-tests were used to identify significant differences in the sample means of distributions for continuous variables (age and BMI) between cases and controls.

### Covariates identified for phenotyping algorithm sample

The following covariates were identified for all cases and controls using structured EMR data: 1. Age at index date (index age); 2. Body mass index (BMI); 3. Sex; 4. Genetically determined ancestry; 5. Nursing home status (y/n); 6. Chemotherapy (y/n); 7. Diabetes mellitus (y/n); 8. Human immunodeficiency virus (HIV) positive status (y/n); 9. Any transplant medications (y/n); 10. Any corticosteroid medications (y/n); and 11. Any medium or high-risk antibiotic exposure (y/n). We used the median BMI record for the age year that matched most closely to the participant’s index age. Nursing home status was determined either by structured data on skilled nursing facility residence, or by mentions of nursing home status in social work and case management notes, as identified by NLP (Supplementary Table S3). We flagged chemotherapy using Current Procedural Terminology (CPT) codes 96400, 96408, 96409, 96411–96425, 96520, and 96530. We flagged participants as having diabetes mellitus if they had at least two of the following three indications: 1. An ICD-CM code from ICD-9-CM 250.* or ICD-10-CM E08-E13.*; 2. Prescriptions for diabetes medications including insulin (Supplementary Table S4); or 3. A hemoglobin A1C (HbA1C) reading > 6.5% or a glucose reading of > 200 mg/dL. Participants were flagged as having HIV infection if they had one instance of ICD-9-CM 042.*, ICD-10-CM B20-B24.* or Z21.*. Patients were flagged as having been exposed to transplant or corticosteroid medications if any medication listed in Supplementary Table S4 was administered outside of the exclusionary time range.

### Genotyping and imputation

Genotypes for all participant samples from eMERGE-I, eMERGE-II and eMERGE-III were imputed using the Michigan Imputation Server^64^. The server uses the Minimac3 algorithm to impute missing genotypes and uses the Haplotype Reference Consortium reference panels^65^ (HRC1.1) as the reference set. The majority of samples from the 13 eMERGE sites were genotyped on the Human 660 Quad (eMERGE-I). Other genotyping platforms included the CytoSNP-850K BeadChip, the OmniExpress chip, the Affymetrix 6.0 array, and the Illumina MEGA among others. In this analysis, variants with an allelic R^2^ ≥ 0.3 and minor allele frequency (MAF) ≥ 0.05 were included. Additional QC filters were applied as described in case–control selection.

### Genetically determined ancestry

The set of ~ 99,000 unique imputed samples was analyzed by Principal Component Analysis (PCA) using the PLINK 2.0 software^66^. Variants with ≥ 0.05 MAF, missingness of ≤ 0.1 and LD-pruned R^2^ threshold of 0.7 were included in the multisample analysis. K-means clustering of Principal Component (PC) 1 and PC2 identified three groups (corresponding to African ancestry, Asian ancestry and European ancestry) was used to find genetically determined ancestry of each sample. Genetically determined and self-identified ancestry were checked for concordance, and samples were ultimately grouped into African ancestry, Asian ancestry, and European ancestry clusters. IBD was calculated for all pairwise sample comparisons using the plink –genome function, and cryptic relatedness between samples was assessed as described in case/control selection.

### GWAS

To identify genetic variants associated with CDI, we performed logistic regression-based association analyses for the case/control curated phenotype using PLINK 1.90^67^. All covariates and genotypes were used in the joint analysis of all participants, whereas the PC1 and PC2 covariates for the African and European ancestry-stratified analyses were derived from ancestry specific PCA analyses. An additive genotypic model of SNV genotypes coded as 0, 1 or 2 copies of the minor allele was used. The regional LD plots of the index SNV were created using the LocusZoom web-based tool^68^. Following the initial stratified analyses, an additional logistic regression-based association analysis was performed in the European sample using the index SNV as a covariate to determine whether this SNV was truly driving the risk association.

### HLA association analyses

Classical HLA alleles were imputed against four ancestry-specific reference panels (African, Asian, European and Hispanic) using the HIBAG software^69^. *HLA-DRB3*, *4* and *5* gene dosages were inferred based on the *HLA-DRB1* alleles present in each individual, as described in Habets et al.^70^. Calls were quality-filtered for a HIBAG posterior probability of > 0.5.

To test for haplotype-specific effects of the most significantly associated SNVs, four overlapping participant subgroups were selected from the European ancestry sample based on the presence of at least one of the following: 1. *DRB3* gene; 2. *DRB4* gene; 3. *DRB5* gene; or 4. any of the above genes in each participant. Haplotype subgroups were further divided into DR15 and DR16 haplotype carriers (stemming from the *DRB5* gene carriers, or DR51 haplotype family), and DRB1*15:01 carriers (stemming from the DR15 haplotype). Logistic regression-based association analysis was performed separately in each haplotype subgroup, using the same covariates described in “Methods: GWAS” for the European ancestry sample.

To test for HLA alleles driving the association, case–control logistic regression-based association analysis was performed in the European ancestry population sample for 276 classical HLA alleles, using the same covariates described in “GWAS” in “Methods” section for the European ancestry sample. The CEU Chromosome 6 LD dataset from the HapMap 3 project was used to assess LD of the most significantly associated SNVs among classical HLA alleles.

### Supplementary Information

Supplementary Information.

Supplementary Table S2.

## Data Availability

The imputed genotype array data and phenotype data used during the current study are available in the database of Genotypes and Phenotypes (dbGaP) under accession number phs001584.v2.p2 (https://www.ncbi.nlm.nih.gov/projects/gap/cgi-bin/study.cgi?study_id=phs001584.v2.p2). Data is available through controlled-access dbGaP Authorized Access requests only. The ClinicalTrials.gov clinical trial registration number for eMERGE Phase III is NCT03394859 (https://clinicaltrials.gov/ct2/show/NCT03394859?term=electronic+medical+records+and+genomics).

## Code availability

Sample code from this study can be publicly accessed at https://github.com/kmuenzen/emerge_cdiff. For more information, please contact the corresponding authors.

## References

[1] Balsells E (2019). Global burden of *Clostridium difficile* infections: A systematic review and meta-analysis. J. Glob. Health.

[2] Kuijper EJ (2006). Emergence of *Clostridium difficile*-associated disease in North America and Europe. Clin. Microbiol. Infect..

[3] McDonald LC, Killgore GE, Thompson A (2005). An Epidemic, toxin gene-variant strain of *Clostridium difficile*. Engl. J. Med..

[4] O’Connor JR, Johnson S, Gerding DN (2009). *Clostridium difficile* infection caused by the epidemic BI/NAP1/027 strain. Gastroenterology.

[5] Aas J, Gessert CE, Bakken JS (2003). Recurrent *Clostridium difficile* colitis: Case series involving 18 patients treated with donor stool administered via a nasogastric tube. Clin. Infect. Dis..

[6] Guo B, Harstall C, Louie T, van Zanten SV, Dieleman LA (2012). Systematic review: Faecal transplantation for the treatment of *Clostridium difficile*-associated disease. Aliment. Pharmacol. Therap..

[7] McDonald LC (2018). Clinical practice guidelines for *Clostridium difficile* infection in adults and children: 2017 Update by the Infectious Diseases Society of America (IDSA) and Society for Healthcare Epidemiology of America (SHEA). Clin. Infect. Dis..

[8] Crobach MJT (2018). Understanding *Clostridium difficile* colonization. Clin. Microbiol. Rev..

[9] Pépin J (2005). Emergence of fluoroquinolones as the predominant risk factor for *Clostridium difficile*-associated diarrhea: A cohort study during an epidemic in Quebec. Clin. Infect. Dis..

[10] de Lalla F (1989). Third generation cephalosporins as a risk factor for *Clostridium difficile*-associated disease: A four-year survey in a general hospital. J. Antimicrob. Chemother..

[11] Bignardi GE (1998). Risk factors for *Clostridium difficile* infection. J. Hosp. Infect..

[12] Fekete T (2010). Concurrent PPIs and antibiotics for incident *C. difficile* infection were associated with increased risk for recurrent infection. Ann. Intern. Med..

[13] Wurfel MM, Hawn TR (2013). Genetic variants associated with susceptibility to *Helicobacter pylori*. JAMA.

[14] Flores J, Okhuysen PC (2009). Genetics of susceptibility to infection with enteric pathogens. Curr. Opin. Infect. Dis..

[15] Ananthakrishnan AN (2013). Genetic risk factors for *Clostridium difficile* infection in ulcerative colitis. Aliment. Pharmacol. Ther..

[16] Apewokin S (2018). Host genetic susceptibility to *Clostridium difficile* infections in patients undergoing autologous stem cell transplantation: A genome-wide association study. Support. Care Cancer.

[17] Shen J (2020). Genetic association reveals protection against recurrence of *Clostridium difficil*e infection with Bezlotoxumab treatment. mSphere.

[18] Jiang Z-D (2006). A common polymorphism in the interleukin 8 gene promoter is associated with *Clostridium difficile* diarrhea. Am. J. Gastroenterol..

[19] Garey KW (2010). A common polymorphism in the interleukin-8 gene promoter is associated with an increased risk for recurrent *Clostridium difficile* infection. Clin. Infect. Dis..

[20] Carey DJ (2016). The Geisinger MyCode community health initiative: An electronic health record-linked biobank for precision medicine research. Genet. Med..

[21] Li J (2021). Variants at the MHC region associate with susceptibility to *Clostridioides difficile* infection: A genome-wide association study using comprehensive electronic health records. Front. Immunol..

[22] McCarty CA (2011). The eMERGE network: A consortium of biorepositories linked to electronic medical records data for conducting genomic studies. BMC Med. Genom..

[23] Buniello A (2019). The NHGRI-EBI GWAS catalog of published genome-wide association studies, targeted arrays and summary statistics 2019. Nucleic Acids Res..

[24] Sherry ST (2001). dbSNP: The NCBI database of genetic variation. Nucleic Acids Res..

[25] Mero I-L (2013). Oligoclonal band status in Scandinavian multiple sclerosis patients is associated with specific genetic risk alleles. PLoS ONE.

[26] Horton R (2004). Gene map of the extended human MHC. Nat. Rev. Genet..

[27] Boyle AP (2012). Annotation of functional variation in personal genomes using RegulomeDB. Genome Res..

[28] Trowsdale J (1995). ‘Both man & bird & beast’: Comparative organization of MHC genes. Immunogenetics.

[29] de Bakker PIW (2006). A high-resolution HLA and SNP haplotype map for disease association studies in the extended human MHC. Nat. Genet..

[30] Machiela MJ, Chanock SJ (2015). LDlink: A web-based application for exploring population-specific haplotype structure and linking correlated alleles of possible functional variants. Bioinformatics.

[31] Chaplin DD (2010). Overview of the immune response. J. Allergy Clin. Immunol..

[32] Kasahara M, Klein D, Vincek V, Sarapata DE, Klein J (1992). Comparative anatomy of the primate major histocompatibility complex DR subregion: Evidence for combinations of DRB genes conserved across species. Genomics.

[33] Penn DJ, Damjanovich K, Potts WK (2002). MHC heterozygosity confers a selective advantage against multiple-strain infections. Proc. Natl. Acad. Sci. U.S.A..

[34] Dunstan SJ (2014). Variation at HLA-DRB1 is associated with resistance to enteric fever. Nat. Genet..

[35] Horn GT, Bugawan TL, Long CM, Manos MM, Erlich HA (1988). Sequence analysis of HLA class II genes from insulin-dependent diabetic individuals. Hum. Immunol..

[36] Wordsworth P (1992). HLA heterozygosity contributes to susceptibility to rheumatoid arthritis. Am. J. Hum. Genet..

[37] Sospedra M (2006). Redundancy in antigen-presenting function of the HLA-DR and -DQ molecules in the multiple sclerosis-associated HLA-DR2 haplotype. J. Immunol..

[38] Yamamoto-Furusho JK, Rodríguez-Bores L, Granados J (2010). HLA-DRB1 alleles are associated with the clinical course of disease and steroid dependence in Mexican patients with ulcerative colitis. Colorectal Dis..

[39] Lambert JC (2013). Meta-analysis of 74,046 individuals identifies 11 new susceptibility loci for Alzheimer’s disease. Nat. Genet..

[40] Mahdi BM (2015). Role of HLA typing on Crohn’s disease pathogenesis. Ann. Med. Surg. (Lond.).

[41] Fogdell A, Hillert J, Sachs C, Olerup O (1995). The multiple sclerosis- and narcolepsy-associated HLA class II haplotype includes the DRB5*0101 allele. Tissue Antigens.

[42] International Multiple Sclerosis Genetics Consortium (2005). A high-density screen for linkage in multiple sclerosis. Am. J. Hum. Genet..

[43] Stürner KH (2019). Is multiple sclerosis progression associated with the HLA-DR15 haplotype?. Mult. Scler. J. Exp. Transl. Clin..

[44] Quandt JA (2012). Myelin basic protein-specific TCR/HLA-DRB5*01:01 transgenic mice support the etiologic role of DRB5*01:01 in multiple sclerosis. J. Immunol..

[45] Enz LS (2020). Increased HLA-DR expression and cortical demyelination in MS links with HLA-DR15. Neurol. Neuroimmunol. Neuroinflamm..

[46] Phelps RG, Rees AJ (1999). The HLA complex in Goodpasture’s disease: A model for analyzing susceptibility to autoimmunity. Kidney Int..

[47] Ooi JD (2013). The HLA-DRB1*15:01-restricted Goodpasture’s T cell epitope induces GN. J. Am. Soc. Nephrol..

[48] Asano T (2017). Effects of HLA-DRB1 alleles on susceptibility and clinical manifestations in Japanese patients with adult onset Still’s disease. Arthritis Res. Ther..

[49] Catanzaro JR (2019). IgA-deficient humans exhibit gut microbiota dysbiosis despite secretion of compensatory IgM. Sci. Rep..

[50] Lycke NY, Bemark M (2017). The regulation of gut mucosal IgA B-cell responses: Recent developments. Mucosal Immunol..

[51] Kubinak JL (2015). MHC variation sculpts individualized microbial communities that control susceptibility to enteric infection. Nat. Commun..

[52] Khan AA (2019). Polymorphic immune mechanisms regulate commensal repertoire. Cell Rep..

[53] Ourth DD (1974). Neutralization of diphtheria toxin by human immunoglobulin classes and subunits. Immunochemistry.

[54] Wilcox MH (2017). Bezlotoxumab for prevention of recurrent *Clostridium difficile* infection. N. Engl. J. Med..

[55] Gupta SB (2016). Antibodies to toxin B are protective against *Clostridium difficile* infection recurrence. Clin. Infect. Dis..

[56] Rees WD, Steiner TS (2018). Adaptive immune response to *Clostridium difficile* infection: A perspective for prevention and therapy. Eur. J. Immunol..

[57] Robertson KD (2005). DNA methylation and human disease. Nat. Rev. Genet..

[58] Do C (2020). Allele-specific DNA methylation is increased in cancers and its dense mapping in normal plus neoplastic cells increases the yield of disease-associated regulatory SNPs. Genome Biol..

[59] Kular L (2018). DNA methylation as a mediator of HLA-DRB1*15:01 and a protective variant in multiple sclerosis. Nat. Commun..

[60] Kirby JC (2016). PheKB: A catalog and workflow for creating electronic phenotype algorithms for transportability. J. Am. Med. Inform. Assoc..

[61] Carrell, D. & Denny, J. *Clostridium difficile Colitis*. https://phekb.org/phenotype/70 (PheKB, 2012).

[62] Carroll KC (2011). Tests for the diagnosis of *Clostridium difficile* infection: The next generation. Anaerobe.

[63] Stanaway IB (2018). The eMERGE genotype set of 83,717 subjects imputed to ~40 million variants genome wide and association with the herpes zoster medical record phenotype. Genet. Epidemiol..

[64] Das S (2016). Next-generation genotype imputation service and methods. Nat. Genet..

[65] Loh P-R (2016). Reference-based phasing using the Haplotype Reference Consortium panel. Nat. Genet..

[66] Chang CC (2015). Second-generation PLINK: Rising to the challenge of larger and richer datasets. Gigascience.

[67] Purcell S (2007). PLINK: A tool set for whole-genome association and population-based linkage analyses. Am. J. Hum. Genet..

[68] Pruim RJ (2010). LocusZoom: Regional visualization of genome-wide association scan results. Bioinformatics.

[69] Zheng, X. *HIBAG: An R Package for HLA Genotype Imputation with Attribute Bagging* (2014). 10.1038/tpj.2013.18 PMC3772955 23712092

[70] Habets THPM (2018). The prevalence of antibodies against the HLA-DRB3 protein in kidney transplantation and the correlation with HLA expression. PLoS ONE.

