# Author Correction: Genetic variation in the human leukocyte antigen region confers susceptibility to *Clostridioides difficile* infection

**DOI:** 10.1038/s41598-023-47359-3

**Published:** 2023-11-15

**Authors:** Kathleen Ferar, Taryn O. Hall, Dana C. Crawford, Robb Rowley, Benjamin A. Satterfield, Rongling Li, Loren Gragert, Elizabeth W. Karlson, Mariza de Andrade, Iftikhar J. Kullo, Catherine A. McCarty, Abel Kho, M. Geoffrey Hayes, Marylyn D. Ritchie, Paul K. Crane, Daniel B. Mirel, Christopher Carlson, John J. Connolly, Hakon Hakonarson, Andrew T. Crenshaw, David Carrell, Yuan Luo, Ozan Dikilitas, Joshua C. Denny, Gail P. Jarvik, David R. Crosslin

**Affiliations:** 1 Department of Biomedical Informatics and Medical Education, University of Washington, Seattle, WA USA; 2 Optum Genomics, UnitedHealth Group, Minnetonka, MN USA; 3 Department of Population and Quantitative Health Sciences, Cleveland Institute for Computational Biology, Case Western Reserve University, Cleveland, OH USA; 4 Department of Genetics and Genome Sciences, Cleveland Institute for Computational Biology, Case Western Reserve University, Cleveland, OH USA; 5 National Human Genome Research Institute, National Institutes of Health, Bethesda, MD USA; 6 Department of Cardiovascular Diseases, Mayo Clinic, Rochester, MN USA; 7 Division of Biomedical Informatics and Genomics, John W. Deming Department of Medicine, Tulane University School of Medicine, New Orleans, LA USA; 8 Department of Medicine, Brigham and Women’s Hospital, Boston, MA USA; 9 Division of Biomedical Statistics and Informatics, Mayo Clinic, Rochester, MN USA; 10 University of Minnesota Medical School, Duluth, MN USA; 11 Center for Human Genetics, Marshfield Clinic Research Foundation, Marshfield, WI USA; 12 Divisions of General Internal Medicine and Preventive Medicine, Northwestern University, Chicago, IL USA; 13 Division of Endocrinology, Metabolism, and Molecular Medicine, Feinberg School of Medicine, Northwestern University, Chicago, IL USA; 14 Department of Biochemistry and Molecular Biology, Center for Systems Genomics, Pennsylvania State University, University Park, PA USA; 15 Division of General Internal Medicine, University of Washington, Seattle, WA USA; 16 Scilligence Corp., Cambridge, MA USA; 17 Public Health Sciences Division, Fred Hutchinson Cancer Research Center, Seattle, WA USA; 18 Center for Applied Genomics, The Children’s Hospital of Philadelphia, Philadelphia, PA USA; 19 Department of Pediatrics, Children’s Hospital of Philadelphia, Philadelphia, PA USA; 20 The Broad Institute of Harvard-MIT, Cambridge, MA USA; 21 Kaiser Permanente Washington Health Research Institute, Seattle, WA USA; 22 Northwestern University Feinberg School of Medicine, Chicago, IL USA; 23 Department of Biomedical Informatics, Vanderbilt University, Nashville, TN USA; 24 Department of Medicine (Medical Genetics), University of Washington Medical Center, Seattle, WA USA

Correction to: *Scientific Reports* 10.1038/s41598-023-45649-4, published online 28 October 2023

In the original version of this Article, Loren Gragert was incorrectly affiliated with ‘Department of Pathology and Laboratory Medicine, Tulane University School of Medicine, New Orleans, LA, USA’. The correct affiliation is listed below:

**Loren Gragert:**

‘Division of Biomedical Informatics and Genomics, John W. Deming Department of Medicine, Tulane University School of Medicine, New Orleans, LA’

The original Article has been corrected.

