# Supplementary material for: Genetic variation in the human leukocyte antigen region confers susceptibility to Clostridioides difficile infection
Source: Sci Rep. 2023 Oct 28;13:18532. doi: 10.1038/s41598-023-45649-4 (PMC10613277; doi:10.1038/s41598-023-45649-4)
Supplement: Supplementary file 1 — Supplementary Information. [file 41598_2023_45649_MOESM1_ESM.pdf]

Title

Genetic variation in the human leukocyte antigen region confers susceptibility to *Clostridioides difficile* infection

Authors

Kathleen Ferar
Taryn O. Hall
Dana C. Crawford
Robb Rowley
Benjamin A. Satterfield
Rongling Li
Loren Gragert
Elizabeth W. Karlson
Mariza de Andrade
Iftikhar J. Kullo
Catherine A. McCarty
Abel Kho
M. Geoffrey Hayes
Marylyn D. Ritchie
Paul K. Crane
Daniel B. Mirel
Christopher Carlson
John J. Connolly
Hakon Hakonarson
Andrew T. Crenshaw
David Carrell
Yuan Luo
Ozan Dikilitas
Joshua C. Denny
Gail P. Jarvik
David R. Crosslin
The electronic Medical Records and Genomics (eMERGE) Network

Supplementary Table S1. *C. difficile* progress note mentions used by the natural language processing algorithm.

**Mentions**
difficile colitis<br>diff colitis<br>dif colitis<br>difficile diarrhea<br>diff diarrhea<br>dif diarrhea<br>difficile infection<br>diff infection<br>dif infection<br>difficile enteritis<br>diff enteritis<br>dif enteritis
*The commonly used abbreviation for clostridiodes/clostridium is the single letter “c”. This is difficult to implement in a word search or dictionary look up and was therefore omitted from the NLP algorithm.

Supplementary Table S2. Class 1 (high risk) and Class 2 (moderate risk) antibiotics, as defined by the eMERGE *C. diff.* phenotyping algorithm.

See Excel spreadsheet.

Supplementary Table S3. Nursing home mentions used by the natural language processing algorithm.

**Generic Names**
NH<br>NSH<br>nursing home<br>SNF<br>skilled nursing facility<br>Hospice<br>NHC
**Proper Names (area specific)**
Cumberland Manor<br>Ida Culver House<br>etc.

Supplementary Table S4. Medications used for case-control exclusion and covariate analysis.

Transplant Medications	Corticosteroids	Diabetes Mellitus Medications
Cellcept<br>munoloc<br>mycophenylate mofetil<br>Tacrolimus<br>fk-506<br>fk5<br>k506<br>tacarolimus<br>tacrolimus hydrate<br>fujimycin<br>lcp-tacro<br>prograf<br>protopic<br>Cyclosporine<br>ciclosporin<br>cyclosporin<br>cyclosporin a<br>gengraf<br>neoral<br>restasis<br>sandimmune<br>sangcya<br>azothioprine<br>azathioprin<br>azathioprine sodium<br>azatioprin<br>azamun<br>azanin<br>azasan<br>ccucol<br>imuran	Cortisone<br>Cortisone Acetate<br>Hydrocortisone<br>Hydrocortisone Sodium Phosphate<br>Hydrocortisone Sodium Succinate<br>Hydrocortisone Acetate<br>Hydrocortisone Cypionate<br>Prednisone<br>Prednisolone<br>Prednisolone Sodium Phosphate<br>Methylprednisolone<br>Methylprednisolone Sodium Succinate<br>Methylprednisolone Acetate<br>Triamcinolone<br>Triamcinolone Acetonide<br>Triamcinolone Diacetate<br>Triamcinolone Hexacetonide<br>Dexamethasone<br>Dexamethasone Acetate<br>Dexamethasone Sodium Phosphate<br>Betamethasone<br>Betamethasone Sodium Phosphate<br>Betamethasone Acetate	Insulin<br>glucagon<br>glucagon-like peptide-1 (GLP-1) receptor agonists<br>biguanides<br>sulfonylurea<br>thiazolidinediones<br>meglitinides<br>biguanides<br>α -glucose inhibitor<br>DPP-4 inhibitors<br>SGLT2 inhibitors<br>Cycloset

Supplementary Figure S1. (Quantile-Quantile) Q-Q plot for logistic regression analysis in the European ancestry sample (n=14,620, λ=1.02). Expected *P*-values from a theoretical χ2-distribution are plotted on the X-axis, and observed *P*-values for each SNV in the logistic regression model are plotted on the Y-axis. The red line represents the null hypothesis that the theoretical and observed *P*-values correspond with one another.

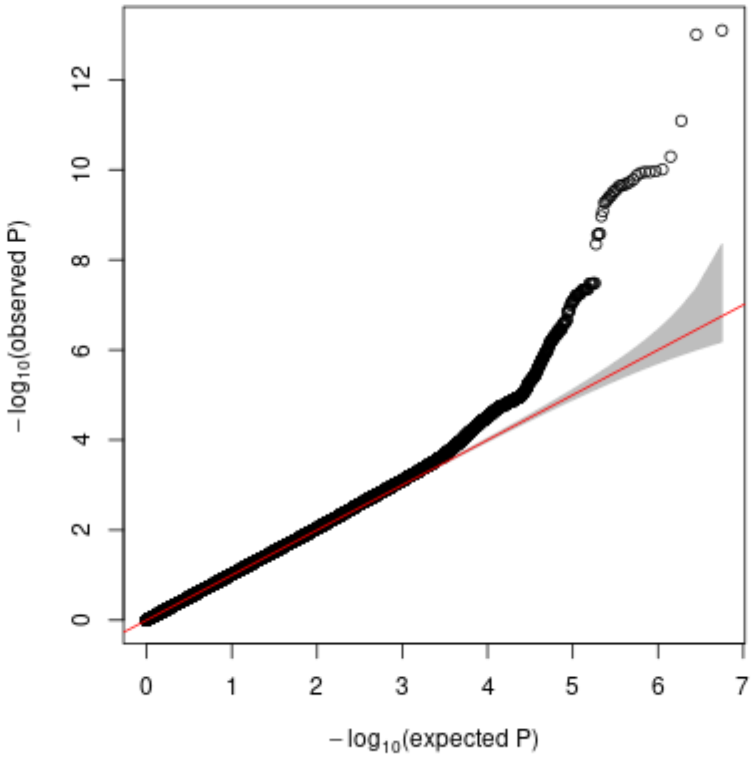

Supplementary Figure S2. Manhattan plot of *P*-values generated using logistic regression analysis in the joint ancestry sample (n=19,861). An additive model was used to assess the disease susceptibility impact of the minor (coded) allele at each position, while controlling for age, BMI, sex, ancestry, nursing home status, chemotherapy, diabetes, HIV, transplant medications, corticosteroids, and medium or high-risk antibiotic exposure as covariates. Genomic coordinates are displayed along the X-axis, and the negative logarithm of logistic regression *P*-values are displayed on the Y-axis. Each dot represents a SNV in the regression model, with associated *P*-values plotted accordingly, while the diamond represents the most significantly associated SNV. The dotted line represents the negative logarithm of the genome-wide significance threshold ($P < 5 \times 10^{-8}$). Colors are used to distinguish between SNVs in adjacent chromosomes.

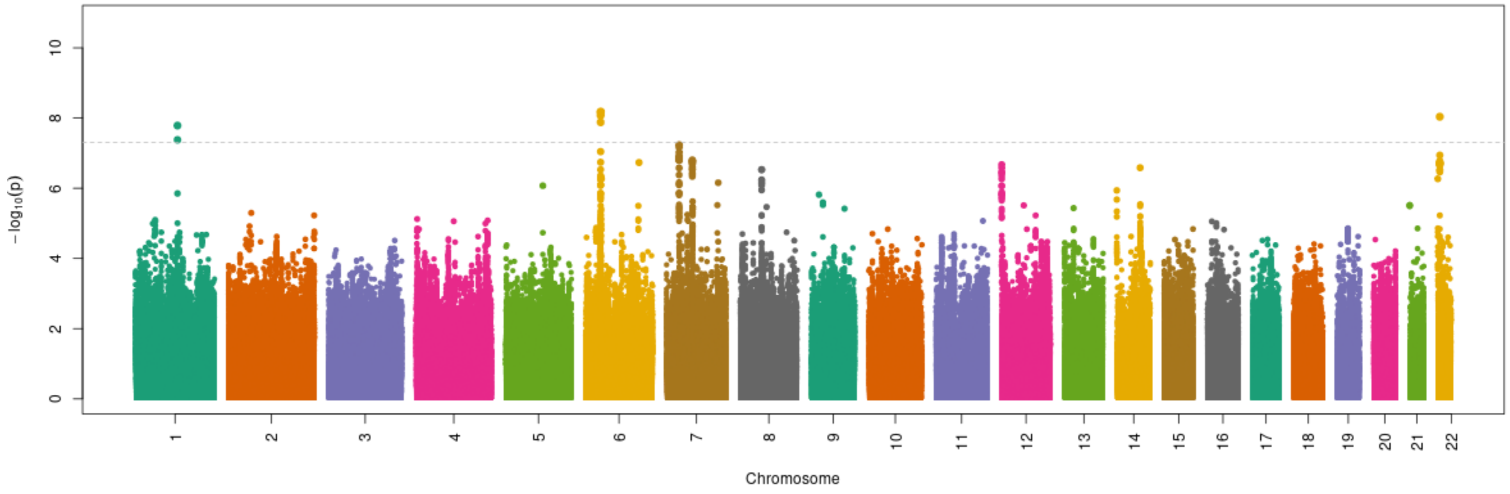

Supplementary Figure S3. Q-Q plot for logistic regression analysis in the joint ancestry sample (n=19,861, λ=1.04). Expected *P*-values from a theoretical χ2-distribution are plotted on the X-axis, and observed *P*-values for each SNV in the logistic regression model are plotted on the Y-axis. The red line represents the null hypothesis that the theoretical and observed *P*-values correspond with one another.

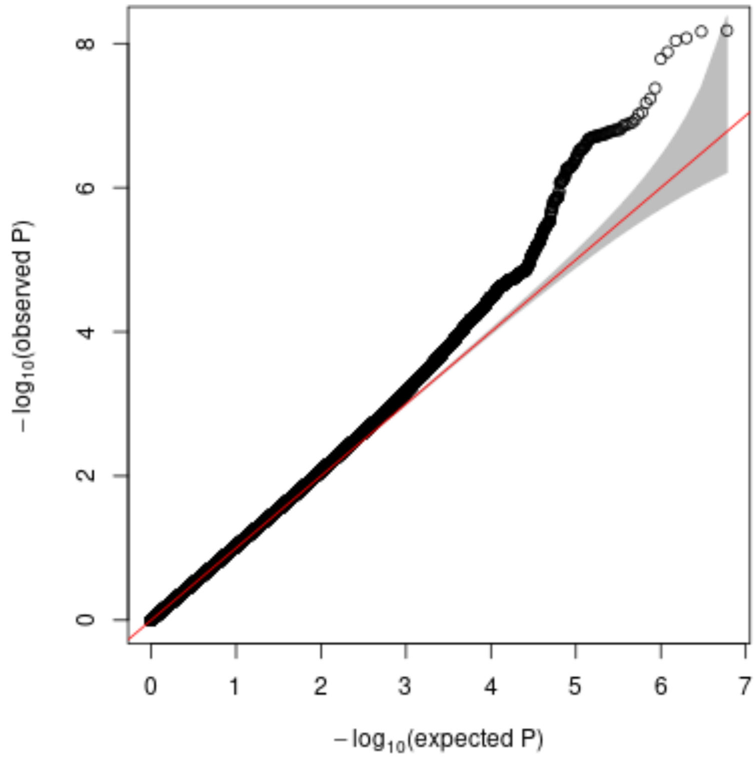

Supplementary Figure S4. Manhattan plot of *P*-values generated using logistic regression analysis in the African ancestry sample (n=3,700). An additive model was used to assess the disease susceptibility impact of the minor (coded) allele at each position, while controlling for age, BMI, sex, ancestry, nursing home status, chemotherapy, diabetes, HIV, transplant medications, corticosteroids, and medium or high-risk antibiotic exposure as covariates. Genomic coordinates are displayed along the X-axis, and the negative logarithm of logistic regression *P*-values are displayed on the Y-axis. Each dot represents a SNV in the regression model, with associated *P*-values plotted accordingly. The dotted line represents the negative logarithm of the genome-wide significance threshold ($P < 5 \times 10^{-8}$). Colors are used to distinguish between SNVs in adjacent chromosomes.

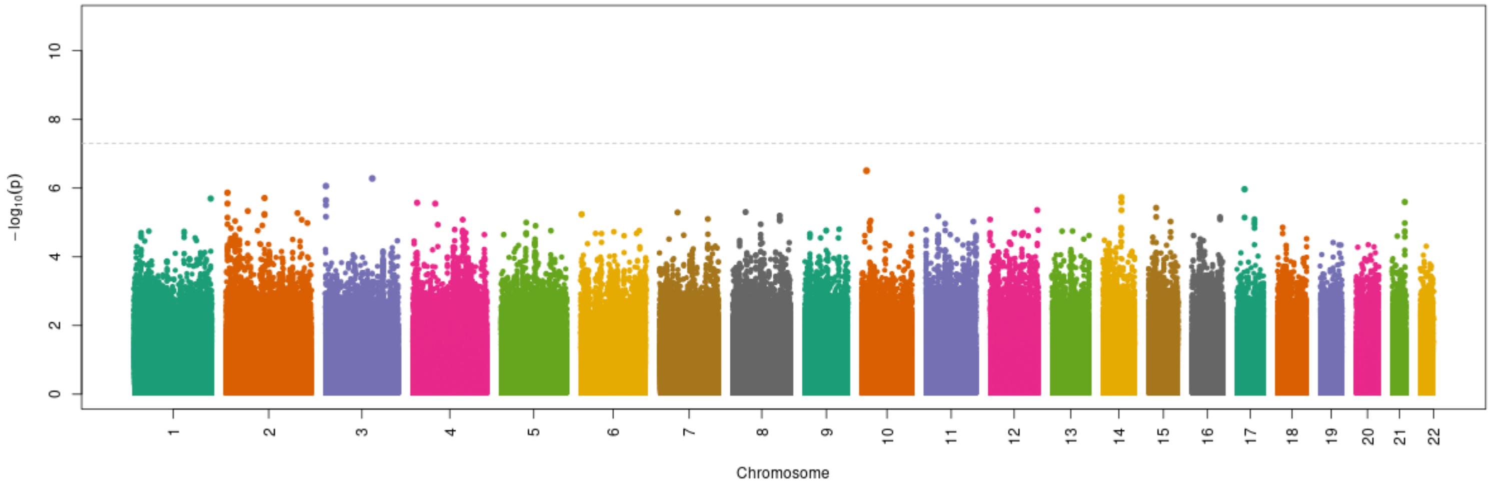

Supplementary Figure S5. Q-Q plot for logistic regression analysis in the African ancestry sample (n=3,700, λ=1.00). Expected *P*-values from a theoretical χ2-distribution are plotted on the X-axis, and observed *P*-values for each SNV in the logistic regression model are plotted on the Y-axis. The red line represents the null hypothesis that the theoretical and observed *P*-values correspond with one another.

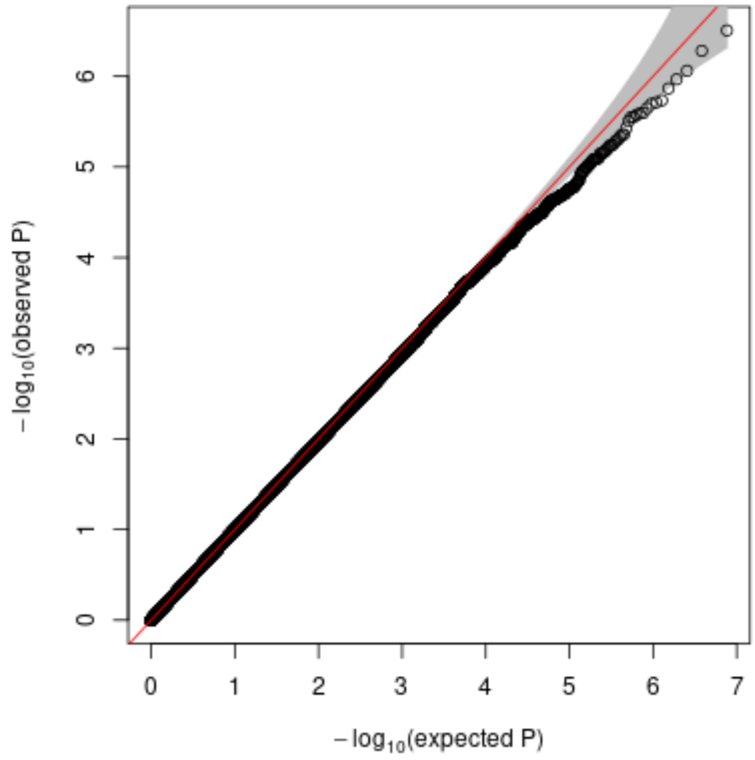

Supplementary Figure S6. Regional linkage disequilibrium (LD) plot of SNVs evaluated in the African-ancestry logistic regression analysis, using the African 1000 Genomes superpopulation as a reference group. Genomic coordinates spanning the HLA-DRB region and surrounding genes are shown on the X-axis in both subplots. Negative logarithms of *P*-values from the African-ancestry logistic regression analysis are shown on the Y-axis in the upper sublot, and annotated gene transcripts are distributed along the Y-axis in the lower subplot. Each dot represents a SNV in the regression model, with associated *P*-values plotted accordingly. SNVs in high LD with reference to the index SNV (rs68148149) are colored in red. The LD plot was generated with the LocusZoom[67] tool using default parameters and the 1000 Genomes Project 2014 AFR reference panel.

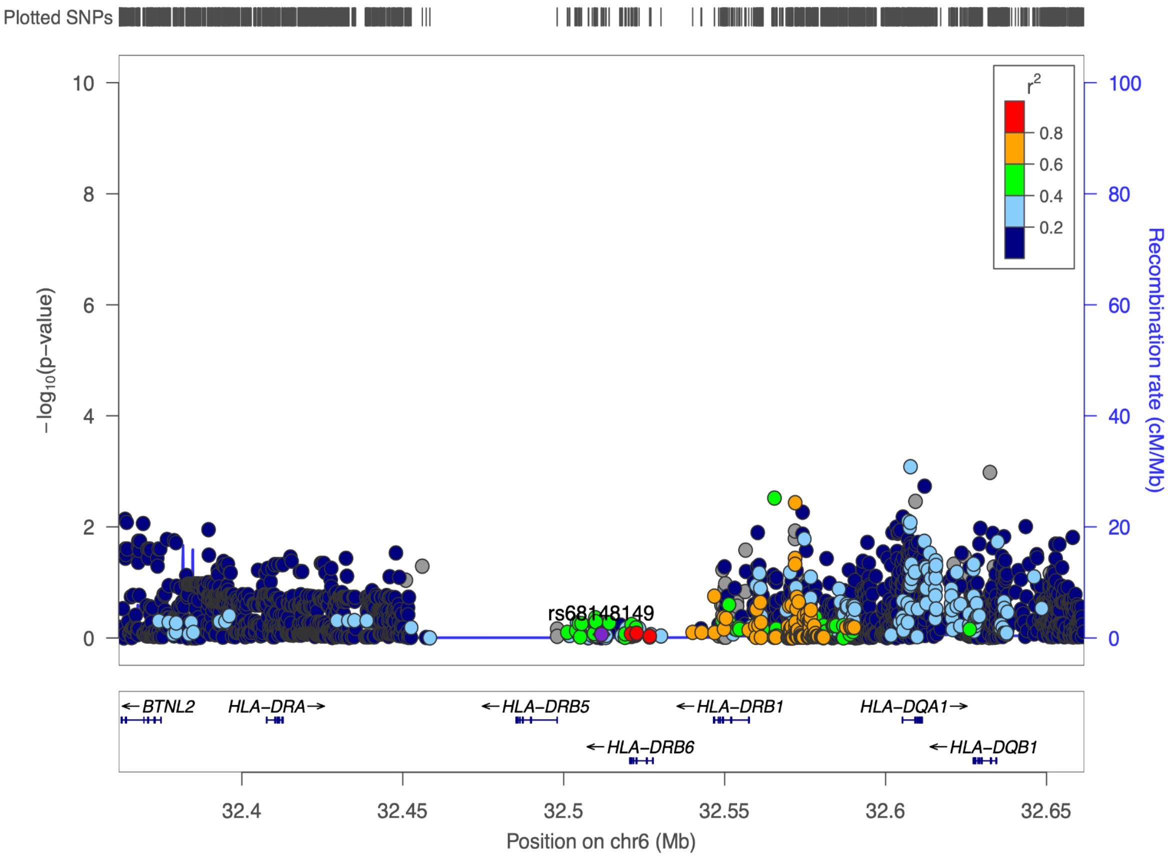

Supplementary Figure S7. Manhattan plot of *P*-values generated using logistic regression analysis in the European ancestry sample (n=14,620), controlling for the index SNV identified in the joint and European-ancestry genome-wide logistic regression analyses (rs68148149). An additive model was used to assess the disease susceptibility impact of the minor (coded) allele at each position, while controlling for the index SNV, age, BMI, sex, ancestry, nursing home status, chemotherapy, diabetes, HIV, transplant medications, corticosteroids, and medium or high-risk antibiotic exposure as covariates. Genomic coordinates are displayed along the X-axis, and the negative logarithm of logistic regression *P*-values are displayed on the Y-axis. Each dot represents a SNV in the regression model, with associated *P*-values plotted accordingly. The dotted line represents the negative logarithm of the genome-wide significance threshold ($P < 5 \times 10^{-8}$). Colors are used to distinguish between SNVs in adjacent chromosomes.

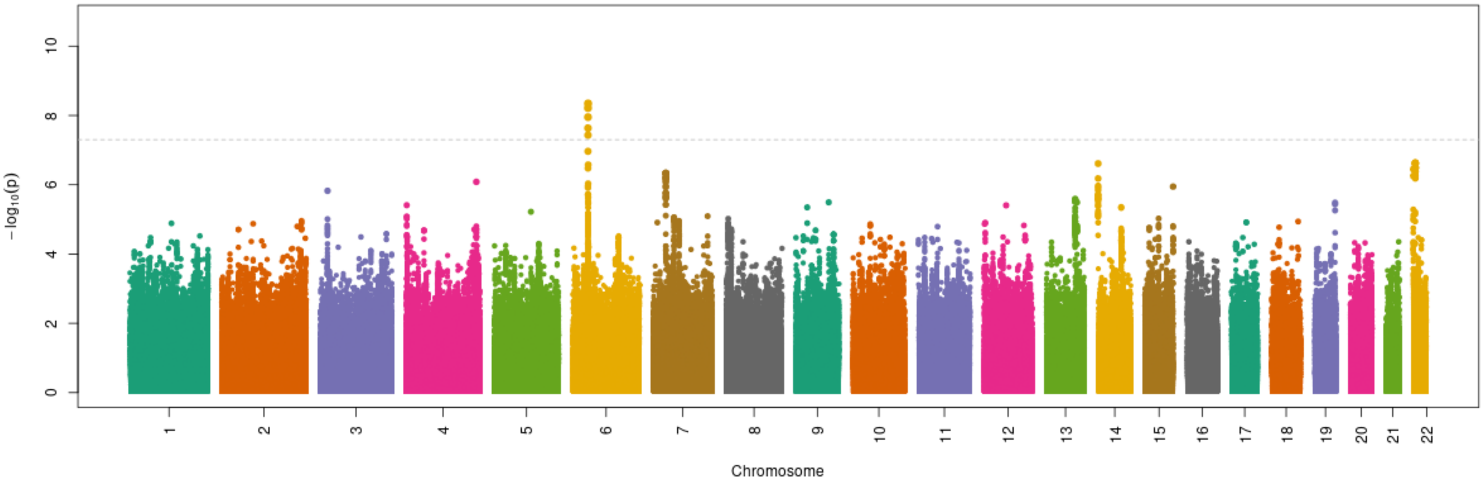

Supplementary Figure S8. Q-Q plot for logistic regression analysis in the European ancestry sample (n=14,620, λ=1.02), controlling for the index SNV identified in the joint and European-ancestry genome-wide logistic regression analyses (rs68148149). Expected *P*-values from a theoretical χ2-distribution are plotted on the X-axis, and observed *P*-values for each SNV in the logistic regression model are plotted on the Y-axis. The red line represents the null hypothesis that the theoretical and observed *P*-values correspond with one another.

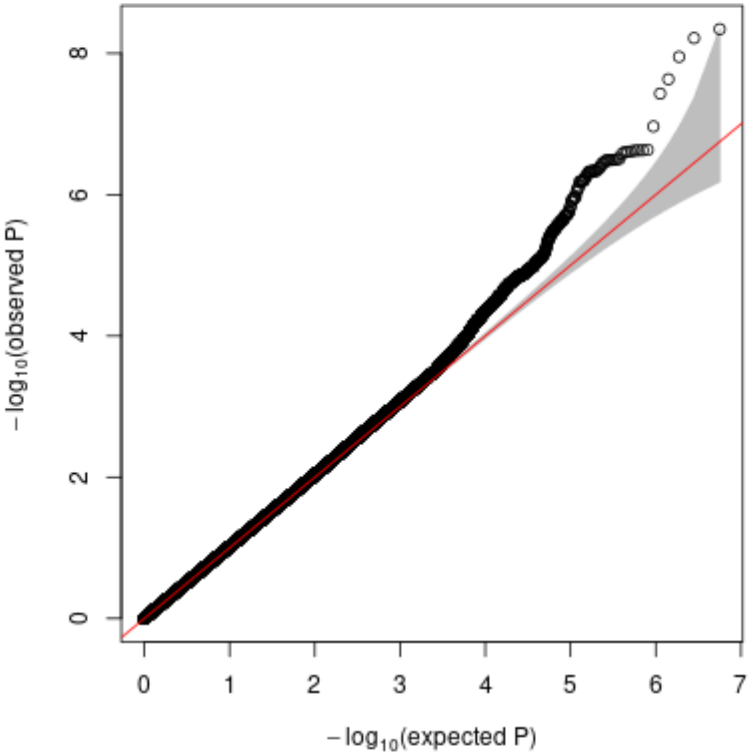

Supplementary Figure S9. Regional Manhattan plot of *P*-values generated using logistic regression analysis of SNVs in the chr6:32400001-32600000 region for 4 participant groups: participants with >=1 copies of the DR51, 52 or 53 haplotype (top left, n=14,291), participants with >=1 copies of the DR51 haplotype (top right, n=4,130), participants with >=1 copies of the DR52 haplotype (bottom left, n=8,887), and participants with >=1 copies of DR53 haplotype (bottom right, n=7,863). An additive model was used to assess the disease susceptibility impact of the minor (coded) allele at each position within each participant group, while controlling for age, BMI, sex, ancestry, nursing home status, chemotherapy, diabetes, HIV, transplant medications, corticosteroids, and medium or high-risk antibiotic exposure as covariates. Genomic coordinates are displayed along the X-axis, and the negative logarithm of logistic regression *P*-values are displayed on the Y-axis of each plot. Each dot represents a SNV in the regression model, with associated *P*-values plotted accordingly. The red line in each plot represents the negative logarithm of the genome-wide significance threshold ($P < 5 \times 10^{-8}$), and the blue line represents a suggestive genome-wide significance threshold ($P < 5 \times 10^{-6}$). Significantly associated SNVs from **Table 3** are colored in green.

DR51(+) or DR52(+) or DR53(+)
(N = 14291)

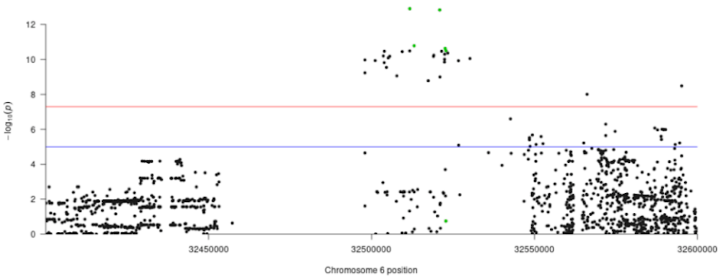

DR51(+), DR52(?), DR53(?)
(N = 4130)

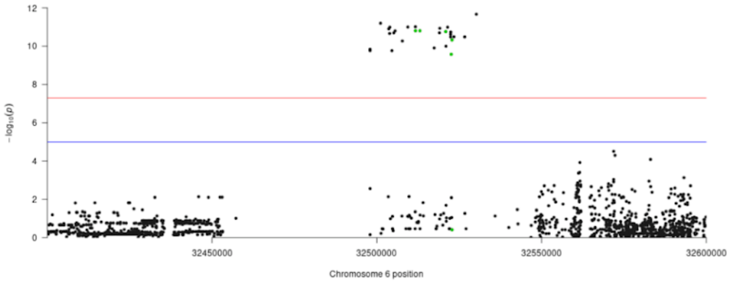

DR51(?), **DR52(+)**, DR53(?)
(N = 8887)

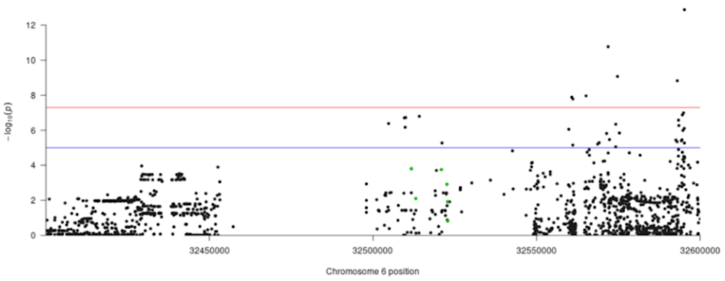

DR51(?), DR52(?), **DR53(+)**
(N = 7863)

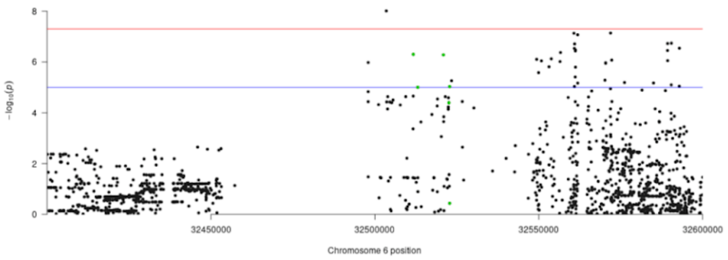

Supplementary Figure S10. Flowchart of regional Manhattan plots of *P*-values generated using logistic regression analysis of SNVs in the chr6:32400001-32600000 region, categorized by the following haplotype subsamples: DR51(+) (n=4130), DR15(+) (n=3791), DR16(+) (n=381), and DRB1*15:01(+) (n=3608). An additive model was used to assess the disease susceptibility impact of the minor (coded) allele at each position within each participant group, while controlling for age, BMI, sex, ancestry, nursing home status, chemotherapy, diabetes, HIV, transplant medications, corticosteroids, and medium or high-risk antibiotic exposure as covariates. Genomic coordinates are displayed along the X-axis, and the negative logarithm of logistic regression *P*-values are displayed on the Y-axis of each plot. Each dot represents a SNV in the regression model, with associated *P*-values plotted accordingly. The red line in each plot represents the negative logarithm of the genome-wide significance threshold ($P < 5 \times 10^{-8}$), and the blue line represents a suggestive genome-wide significance threshold ($P < 5 \times 10^{-6}$). Significantly associated SNVs from **Table 3** are colored in green.

DR51(+), DR52(?), DR53(?)
(N = 4130)

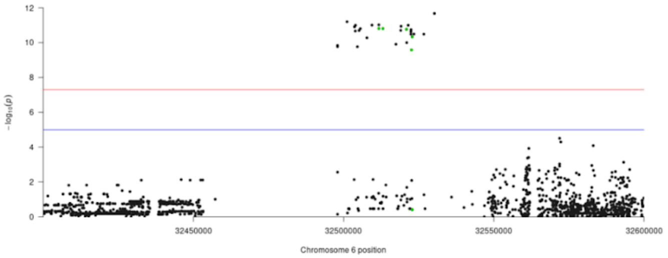

DR15(+), DR16(?), DR52(?), DR53(?)
(N = 3791)

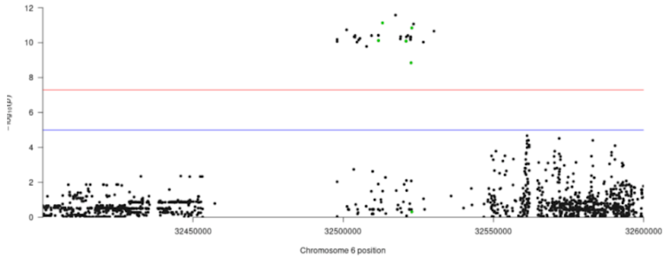

DR15(?), **DR16(+)**, DR52(?), DR53(?)
(N = 381)

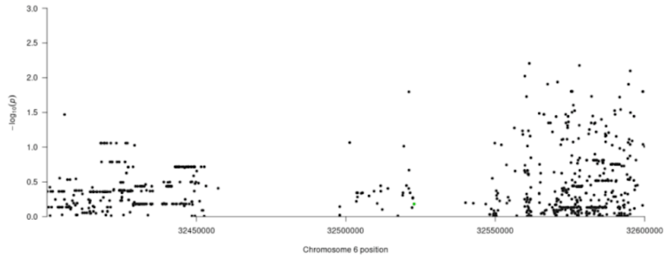

DRB1*15:01(+), DRB1*15:02(?), DR16(?),
DR52(?), DR53(?)
(N = 3608)

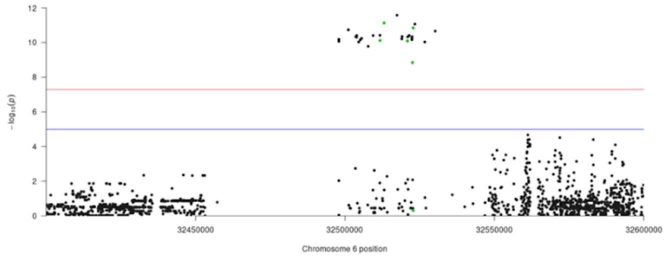

Supplementary Figure S11. Relational flowchart of the *HLA-DRB* haplotypes identified in the eMERGE *C. diff.* cohort.

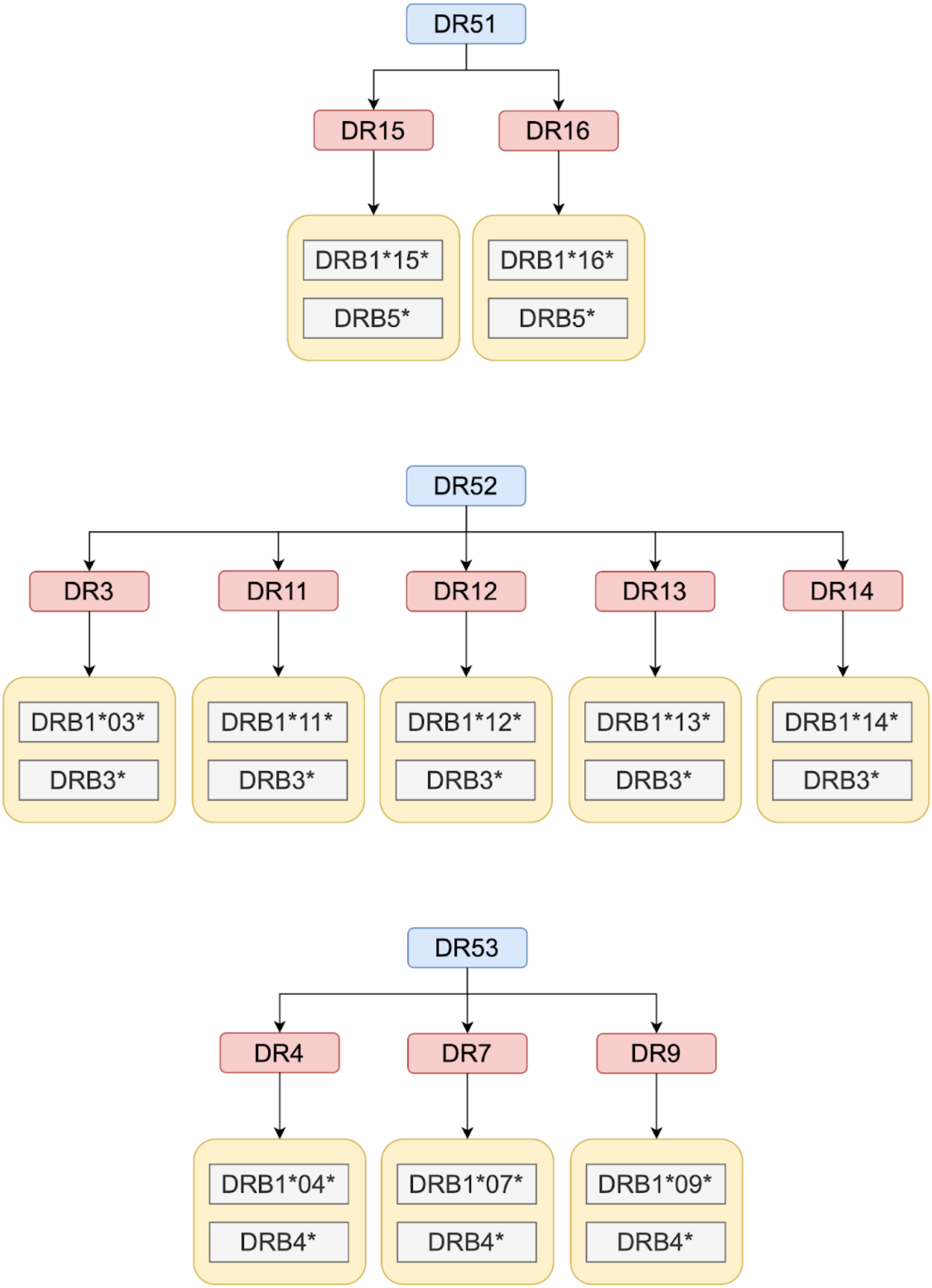

Supplementary Figure S12. Flowchart of coding allele frequencies (CAFs) of the index SNV identified in the joint and European-ancestry genome-wide logistic regression analyses (rs68148149) in different HLA-DR haplotype-enriched groups (DR51, DR52, and DR53).

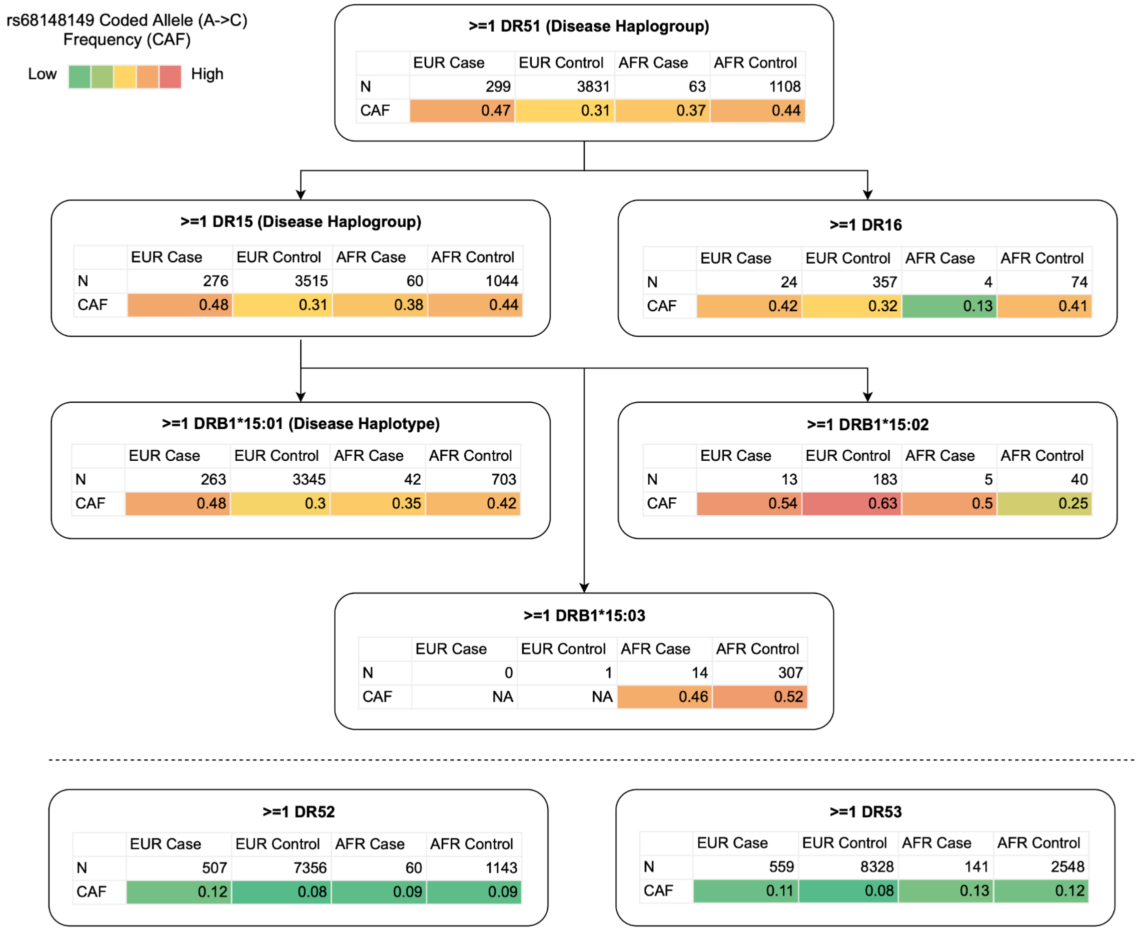

>=1 DR51 (Disease Haplogroup)

	EUR Case	EUR Control	AFR Case	AFR Control
N	299	3831	63	1108
CAF	0.47	0.31	0.37	0.44

>=1 DR15 (Disease Haplogroup)

	EUR Case	EUR Control	AFR Case	AFR Control
N	276	3515	60	1044
CAF	0.48	0.31	0.38	0.44

>=1 DR16

	EUR Case	EUR Control	AFR Case	AFR Control
N	24	357	4	74
CAF	0.42	0.32	0.13	0.41

>=1 DRB1*15:01 (Disease Haplotype)

	EUR Case	EUR Control	AFR Case	AFR Control
N	263	3345	42	703
CAF	0.48	0.3	0.35	0.42

>=1 DRB1*15:02

	EUR Case	EUR Control	AFR Case	AFR Control
N	13	183	5	40
CAF	0.54	0.63	0.5	0.25

>=1 DRB1*15:03

	EUR Case	EUR Control	AFR Case	AFR Control
N	0	1	14	307
CAF	NA	NA	0.46	0.52

>=1 DR52

	EUR Case	EUR Control	AFR Case	AFR Control
N	507	7356	60	1143
CAF	0.12	0.08	0.09	0.09

>=1 DR53

	EUR Case	EUR Control	AFR Case	AFR Control
N	559	8328	141	2548
CAF	0.11	0.08	0.13	0.12
